# The genome sequence of the outbreeding globe artichoke constructed *de novo* incorporating a phase-aware low-pass sequencing strategy of F_1_ progeny

**DOI:** 10.1038/srep19427

**Published:** 2016-01-20

**Authors:** Davide Scaglione, Sebastian Reyes-Chin-Wo, Alberto Acquadro, Lutz Froenicke, Ezio Portis, Christopher Beitel, Matteo Tirone, Rosario Mauro, Antonino Lo Monaco, Giovanni Mauromicale, Primetta Faccioli, Luigi Cattivelli, Loren Rieseberg, Richard Michelmore, Sergio Lanteri

**Affiliations:** 1 DISAFA, Plant Genetics and Breeding, University of Torino, Grugliasco, Italy; 2 The Genome Center, University of California, Davis, CA, USA; 3 Dipartimento di Agricoltura, Alimentazione e Ambiente (Di3A), University of Catania, Catania, Italy; 4 Consiglio per la ricerca in agricoltura e l’analisi dell’economia agraria, Genomics Research Centre, Fiorenzuola d’Arda, Italy; 5 University of British Columbia, Vancouver, BC, Canada

## Abstract

Globe artichoke *(Cynara cardunculus* var. *scolymus)* is an out-crossing, perennial, multi-use crop species that is grown worldwide and belongs to the Compositae, one of the most successful Angiosperm families. We describe the first genome sequence of globe artichoke. The assembly, comprising of 13,588 scaffolds covering 725 of the 1,084 Mb genome, was generated using ~133-fold Illumina sequencing data and encodes 26,889 predicted genes. Re-sequencing (30×) of globe artichoke and cultivated cardoon (*C. cardunculus* var. *altilis*) parental genotypes and low-coverage (0.5 to 1×) genotyping-by-sequencing of 163 F_1_ individuals resulted in 73% of the assembled genome being anchored in 2,178 genetic bins ordered along 17 chromosomal pseudomolecules. This was achieved using a novel pipeline, SOILoCo (Scaffold Ordering by Imputation with Low Coverage), to detect heterozygous regions and assign parental haplotypes with low sequencing read depth and of unknown phase. SOILoCo provides a powerful tool for *de novo* genome analysis of outcrossing species. Our data will enable genome-scale analyses of evolutionary processes among crops, weeds, and wild species within and beyond the Compositae, and will facilitate the identification of economically important genes from related species.

Globe artichoke (*Cynara cardunculus* var. *scolymus* L.) is an out-crossing perennial vegetable crop that is native to the Mediterranean Basin and grown worldwide. Its production has risen from about 1.5 Mt in 1993 to 1.8 Mt in 2013 (FAOSTAT, 2013) with a gross production value of about US$600 million. Italy, which harbours the richest primary gene pool of cultivated globe artichoke, is the top producing country, but globe artichoke cultivation has spread to other Mediterranean countries and in recent years to the Americas and China. In addition to globe artichoke, the *C. cardunculus* complex includes the cultivated cardoon (var. *altilis* DC) and the wild cardoon [var. *sylvestris* (Lamk) Fiori]. The cultivated forms have evolved independently from the wild cardoon in the Mediterranean Basin with selection for different plant characteristics^1^,^2^. The two cultivated and the wild forms are fully cross-compatible. The primary product of globe artichoke is the immature inflorescence (compound head or *capitulum*), whose inner bracts and fleshy receptacle are consumed as a fresh, preserved or frozen delicacy; as are other members of the *C. cardunculus* complex, it is also a source of bio-active compounds, such as antioxidant phenolics, sesquiterpene lactones, and inulin^3^,^4^,^5^ and can be exploited for the production of lignocellulosic biomass and seed oil for both edible and biofuel purposes^6^,^7^,^8^.

*C. cardunculus* is a diploid species (2n = 2× = 34) with a medium-sized genome estimated by flow cytometry to be 1.07 Gb^9^. The few genomics data currently available for this crop include a restriction site associated DNA sequencing (RADseq) genome analysis^10^ and RNAseq data, which provided a reference transcriptome of 38 K unigenes^11^. In addition, several *C. cardunculus* genetic maps have been developed based on a two-way pseudo-testcross approach and QTL analyses for key breeding traits have been conducted^12^,^13^,^14^,^15^. The sequence of the chloroplast genome has recently been reported^16^.

*C. cardunculus* is a member of the Compositae (a.k.a. Asteraceae), which is the largest and one of the most ecologically successful botanical families^17^. The family most likely originated in South America (30 to 100 Mya)^18^. Rapid diversification of the Compositae in the mid-Eocene (40 Mya) is associated with a polyploidization event near the base of the family^19^ and it has been conjectured that the base chromosome number for the family is nine^20^. Of the Compositae crop genomes, the 2.7 Gb lettuce genome (http://lgr.genomecenter.ucdavis.edu; GenomeProject ID: PRJNA68025) and the 3.5 Gb sunflower genome (http://www.sunflowergenome.org; GenomeProject ID: PRJNA64989) have been released; however, neither genome has been formally published. The only published sequence of a Compositae species is that of the 335 Mb genome of horseweed (*Conyza canadensis*)^21^. However, the sequence scaffolds of horseweed have not been placed into linkage groups or integrated into pseudomolecules, so its genome cannot be used for syntenic analyses. Beyond the Compositae, the genomes of several Asterid species (potato^22^; tomato^23^, pepper^24^,^25^ and eggplant^26^,^27^) have been recently sequenced; thus, the availability of the globe artichoke genome adds to our understanding of ancestral genome duplications^19^ and contributes insights into the evolution of the Compositae^20^.

Understanding of the genome structure of globe artichoke provides the foundation for the molecular deciphering of complex traits, the establishment of gene orthology and the study of synteny across the family to strengthen community research. Short-read sequencing technologies offer a cost-effective opportunity for non-model species to gain access to the genomic era. However, chromosome-scale assembly of genomes remains an endeavor that is constrained by costs and limited analytical toolkits^28^,^29^. We generated the reference genome for globe artichoke using a clone with low residual heterozygosity. To assign scaffolds to genetic bins ordered along chromosomal linkage groups, we implemented a novel pipeline named SOILoCo - Scaffold Ordering by Imputation with Low Coverage. This utilizes low coverage (<1×) whole genome shotgun genotyping-by-sequencing of an F_1_ progeny to reliably identify heterozygous loci as well as segregation data for the haplotypes derived from the heterozygous parental lines. Such haplotypes do not need to be present in the reference sequence. The globe artichoke sequence provided information on the overall genome organization and gene content, along with structural and functional annotation. We also inferred the timings of speciation, whole genome duplication and expansion of mobile elements.

## Results

### Genome sequencing and assembly

The Illumina Hiseq2000 platform was used for whole-genome shotgun sequencing of the globe artichoke clone designated ‘2C’. This clone had been obtained by three cycles of selfing (S_3_) and had a residual heterozygosity of approximately 10%, as assessed by analysis of 115 SSR markers^30^,^31^. DNA was extracted from young leaves and one paired-end and two mate-pair libraries generated, consistent with requirements of the ALLPATHS-LG assembly software^32^. The 100 + 100 bp reads of the paired-end library that had an average insert size 170 bp produced 67 Gb of raw data (equivalent to an estimated genome coverage of 62×), with a 96% efficiency in generating 3’-overlaps with an average overlap of 26 bp. The sequencing of the two mate-pair libraries, with average effective insert sizes of 2.5 Kb and 5.5 Kb, produced 56 Gb and 7 Gb of raw data respectively (corresponding to 52× and 6.5× genome coverages). After trimming of sequencing adapters and removal of low-quality bases, a total of 133.7 Gb were available for assembly (Table 1). The analysis of K-mer spectra confirmed the limited residual heterozygosity of the 2C breeding line; this was markedly lower than the levels detected in the parental genotypes (globe artichoke and cultivated cardoon) of our mapping population (see below; Supplementary Fig. S1). Based on K-mer statistics and sequencing depth^33^, the globe artichoke genome size was estimated to be 1,084 Mb, in close agreement with the previous estimation by flow cytofluorimetry^9^. The error-correction routine implemented in ALLPATHS-LG resulted in 87.5% of the filtered paired-end reads being used in the assembly. The 50% of the *de novo* assembly (*N*_50_) was included in 10,596 contigs of 17.5 Kb or larger, with 32% G + C content (Table 2). The contigs were assembled into 13,588 scaffolds (≥1,000 bp), with a *N*_50_ of 1,408 and a *L*_50_ equal to 125.9 Kb. The final assembly spanned approximately 724.7 Mb. The longest scaffold was 1.5 Mb and the median gap size within a scaffold was 660 bp. Scaffolds were finally filtered to remove chloroplast sequences (Supplementary information).

The assembly was compared to the available UniGene set^11^ to assess the coverage of the *C. cardunculus* gene space; 37,018 of the 38,726 unigenes (95.6%) aligned to the assembled genome (Supplementary Methods and Results; Supplementary Table S1a). Unaligned ESTs were analysed by BLASTx as in^34^ against Viridiplantae proteins (Refseq); 675 (69%) of these had hits. These unigenes (1.7% of all available unigenes) can contribute to a hypothetical total of 27,564 gene models, which would reflects a predicted proportion of non-assembled genes up to 2.5%. The genomic draft assembly was also surveyed for the presence of 248 complete conserved eukaryotic genes (CEGs^35^) of which more than 85% were mapped (Supplementary Table S1b). The inclusion of partial matches increased the percentage of mapped CEGs to 96%. While the yield of complete CEGs increased from less conserved (Group 1–80.3%) to highly conserved genes (Group 4–93.85%), the coverage obtained with partial alignments was similar among the groups (95.45% to 98.46%; Supplementary information). This suggests that most of the missing complete alignments were due to sequence divergence and thus, it can be concluded that at least 95% of the gene space was captured in our genome assembly.

### A pipeline for anchoring scaffolds using low-coverage data

**Figure 1:**
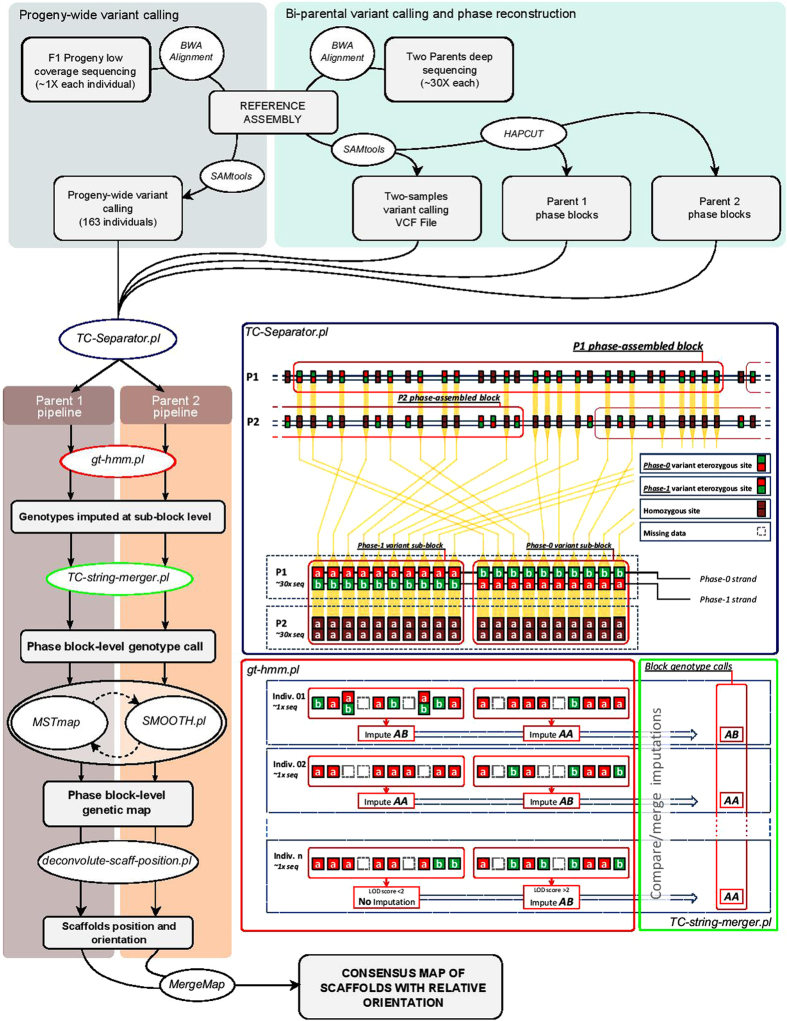
Workflow schema for the SoiLoCo pipeline used to anchor the *C. cardunculus* scaffolds in chromosomal pseudomolecules. Alignments of parental reads to the draft scaffolds were used to (i) identify potential heterozygous test-cross sites and (ii) to compute haplotype phases in both parents (P1 and P2). A multi-sample VCF file of all the progeny was then processed to identify informative heterozygous sites based on parental SNPs and the phase of haplotype blocks (TC-separator.pl, blue box). This assigned the sites according to which phase (i.e. homologous chromosomes) they are expected to segregate in. Subsequently, an HMM-based algorithm was used to impute the most likely genotypes of each haplotype block segregating in the progeny (gt-hmm.pl, red box). A LOD score was also calculated to permit filtering of ambiguous imputations. Genotype imputation from the two alternative segregating phases were then summarized; when there was a discordant call between phases, a majority rule was applied and the highest LOD score for each segregating haplotype used to impute the most likely genotype (TC-string-merger.pl, green box). After grouping markers, linkage maps were generated for each parent using reiterative ordering with the MSTmap software (http://alumni.cs.ucr.edu/~yonghui/mstmap.html) and error correction using a Perl implementation of SMOOTH algorithm^37^. Maps were finally merged to generate a consensus map and to maximize the resolution of the order and orientation of scaffolds in chromosomal pseudomolecules.

Linkage analysis of an F_1_ population of 163 individuals, obtained by crossing globe artichoke C3 as the female parent and cultivated cardoon Alt41 as the male parent, was performed to identify the genetic position of sequence scaffolds and to generate chromosomal pseudomolecules. To achieve this, we developed and implemented a novel method for genotyping-by-sequencing individuals of an F_1_ progeny from low-pass sequencing data combined with conventional deep sequencing of the heterozygous parents, whose linkage phase was unknown. The method relies on the *de novo* identification of parental allelic phases (haplotype blocks), with no reliance on haplotypes being shared with the reference sequence assembly. The pipeline, whose workflow is depicted in Fig. 1, was named SOILoCo (Scaffold Ordering by Imputation with Low Coverage). It requires: (i) a draft assembly of a reference genome; (ii) whole-genome sequencing of each parent to a coverage of at least 20× (being permissive of haplotype reconstruction with HAPCUT; genomes with lower SNP frequencies may require larger fragments to obtain phase calls); and (iii) whole-genome sequencing of their segregating progenies to a coverage as low as 0.5× each. Illumina reads of parental lines were first mapped to the reference sequence assembly in order to identify the positions of robust heterozygous SNPs. The haplotype blocks of each parental genome sequence were subsequently built using the HAPCUT algorithm^36^. SNP polymorphisms were then called with low-confidence for each progeny individual based on low sequence coverage. A set of custom Perl scripts was then applied in order to: (i) catalogue SNP sites expected to segregate in a test-cross fashion; (ii) split the two segregating haplotypes for each parent (i.e. segregating SNP sites from either one or the other homologous chromosomes, phase-0 and phase-1 in Fig. 1); (iii) recover SNP calls for each individual in any given haplotype block/segregating phase; (iv) invoke an Hidden Markov Model (HMM) engine to infer a haplotype block-wide genotype call (Supplementary information); and finally, (v) summarize data and generate a dataset of genotypes for each individual.

**Figure 2:**
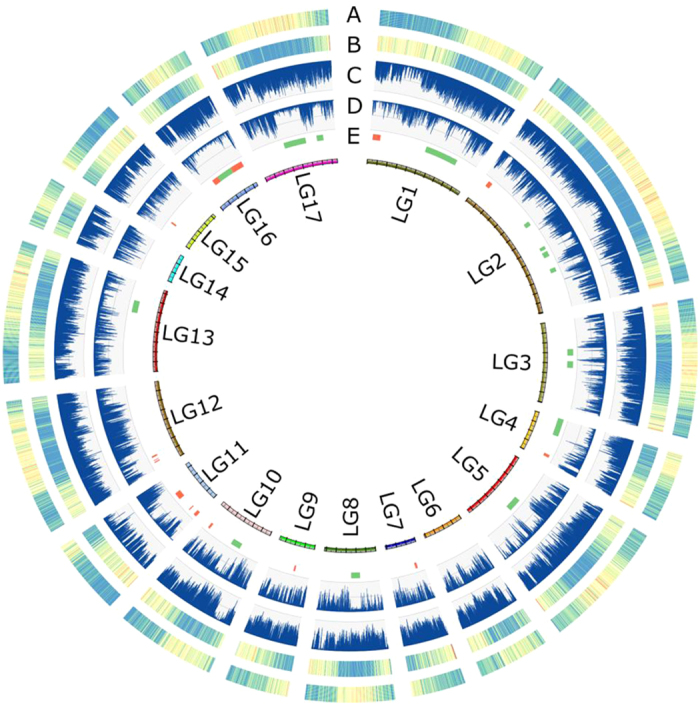
Characteristics of the globe artichoke genome. **(a)** Heat map of repeat density in the reference genome generated from globe artichoke 2C (blue, low; orange, high density); **(b)** Heat map of gene density in the reference genome; **(c)** density of heterozygous SNPs in the globe artichoke parental genotype, C3; **(d)** density of heterozygous SNPs in the cultivated cardoon parental genotype, Alt41; **(e)** extended homozygous regions in cultivated cardoon: the color of the boxes indicate whether they occur in repeat-rich regions (green) or gene-rich regions (red).

**Figure 3:**
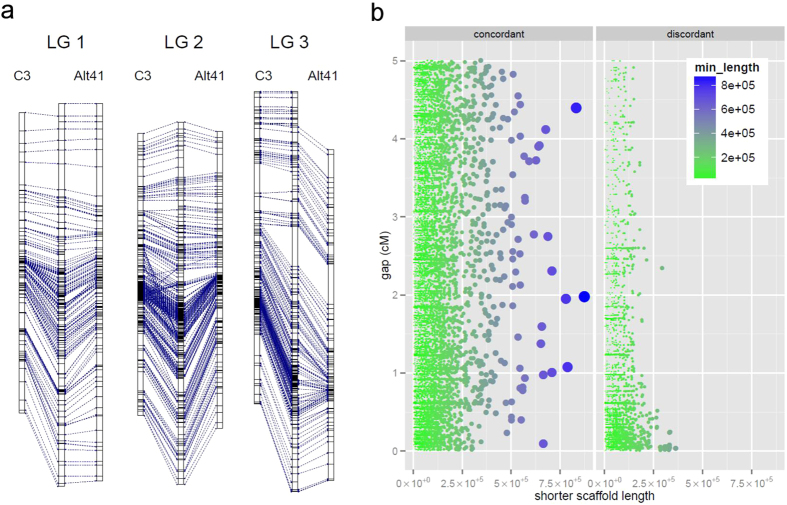
Anchoring the globe artichoke reference genome to the genetic maps and analysis robustness for each map. **(a)** Alignments of parental maps with the inferred consensus map in the middle for the first three *C. cardunculus* linkage groups. **(b)** Distribution of sizes (x axis) and genetic distances (y axis) for every possible pair of scaffolds separated by up to 5 cM when comparing the two parental maps. Pairs are shown as concordant (left) if their relative genetic position is maintained in both maps; discordant (right) if it is not. Scaffold size is reported as the smaller size of the two scaffolds in a pair (size and color of dots emphasize the scaffold size).

Illumina sequencing of the two parental genotypes yielded a total of 520 M (52.0 Gb) and 467 M (46.8 Gb) raw reads, corresponding to a coverage of about 48× and 43× for C3 and Alt41, respectively (Supplementary Table S2). After read trimming and mapping to the reference genome, 5,828,006 and 3,092,899 heterozygous SNP sites were called in the two parental genotypes. In the C3 genotype of globe artichoke, *de novo* haplotype blocks assembly allowed us to compute a total of 192,938 blocks, corresponding to 364 Mb (L_50_: 4,772 bp, N_50_: 19,921) and an average density of 1 SNP per 59 bp. In cultivated cardoon parent Alt41, 122,570 haplotype blocks were successfully identified, corresponding to 187 Mb (L_50_: 4104 bp, N_50_: 12,001) and an average density of 1 SNP per 59 bp. The identical SNP frequency of the parental haplotype blocks indicates that SNP-dense regions contributed most in extending haplotype blocks, while similar contiguities is consistent with our observation that the lower frequency of heterozygous SNP sites in Alt41 is due to long homozygous segments in the genome (Fig. 2) rather than due to a genome-wide reduced level of polymorphism. After the extraction of candidate SNP sites segregating in a test cross fashion (TC-SNPs), 2,128,486 were retained in globe artichoke C3, which were scattered across 138,608 haplotype blocks; while in cultivated cardoon Alt41 1,356,998 were retained, which covered 78,458 haplotype blocks. By invoking the HMM engine and combining the complementary segregation information from either phase-0 or phase-1 polymorphic sites, we retrieved genotype calls for phased haplotype blocks with a minimum calling rate of 70%. Since this F_1_ mapping population had previously been used for linkage mapping in which no extensive segregation distortion had been observed^14^, we filtered out blocks with Χ^2^ test p-value < 0.03. The pseudo-testcross segregation analysis for C3 generated genotype calls for 23,381 blocks, representing 4,234 unique scaffolds, while analysis of Alt41 provided 15,262 genotyped blocks for 2,779 scaffolds; 1,677 mapped scaffolds were shared between the C3 and Alt41 datasets. Using the two-way pseudo-testcross approach^15^, an independent linkage analysis was carried out for each parent, defining 1,157 and 1,497 genetic bins for the globe artichoke C3 and the cultivated cardoon Alt41 maps, respectively. This anchored 445 Mb of the assembly as 23,366 markers in C3 and 358 Mb as 15,227 markers in Alt41. Correction of putative missed calls by a Perl implementation of the SMOOTH algorithm^37^ and subsequent markers re-ordering was iterated twice and resulted in final maps spanning 1,460 cM in C3 and 1,748 cM in Alt41, with an average inter-marker distance of 1.28 cM and 1.18 cM, respectively. Maps were highly co-linear (Fig. 3a) and the generation of a consensus map provided mutual disambiguation of positions within genetic bins. The final dataset comprised of 2,178 distinct genetic anchors ordered along 17 chromosomal pseudo-molecules (Supplementary Table S3) that contained 5,322 scaffolds spanning 526 Mb. Of these, 936 scaffolds (210 Mb) were oriented by correlating the genetic position of each marker within a given scaffold along with their physical coordinates. Chromosomal pseudomolecules were numbered so as to be consistent with previously published linkage groups (Supplementary Table S3).

In order to assess the robustness of our method for genetically anchoring *de novo* assembled scaffolds without the availability of a validated reference genome, we exploited the availability of the two maps generated for each parent. To do this, we made pairwise comparisons of scaffold doublets separated by 5 cM or less. Doublets were considered concordant when the two scaffolds maintained the same reciprocal position across the two maps. This analysis demonstrated that 23,558 pairs of scaffolds (87%) were collinear, while only 3,480 had discordant positions. Furthermore, when only pairs of scaffolds longer than 100 Kb were considered, the ratio of concordant pairs increased to 94% (Fig. 3b). The discordant pairs may have been due to: (i) small differences in genome organization of the two taxa, or (ii) errors in the genotyping process caused by a lack of informative SNP sites or possibly by the presence of confounding false SNPs resulting from repetitive regions which were not filtered out from the alignment.

### Gene space

The annotation pipeline yielded 26,889 gene models (and a total of 27,121 predicted transcripts, Table 3), of which 23,895 were located in the chromosomal pseudomolecules and only 2,994 were unmapped (Table 4). Gene models covered a total of 146.5 Mb, of which 38 Mb were exons, with median length of 141 bp, containing 32.8 Mb of coding sequences with a median length of 127 bp; the remainder comprised of introns with median length of 334 bp and untranslated regions (Table 3, and Supplementary Fig. S2). Figure 2 shows gene-rich regions along with repeat-dense regions and suggests the likely locations of the centromeric regions.

Out of the 27,121 predicted proteins, 19,554 (72%) were functionally annotated. Protein clustering resulted in 3,724 clusters with 21,850 sequences (80.5% of the total number of predicted proteins). Top 15 groups by size are presented in Supplementary Table S4. To obtain an overview of the entire genome, protein annotations were counted across all the groups. The most common functionally annotated predicted proteins in globe artichoke had a protein kinase domain (primarily serine/threonine kinases and tyrosine kinases), followed by the Zinc finger domain and the Leucine Rich Repeats (Supplementary Table S5).

**Figure 4:**
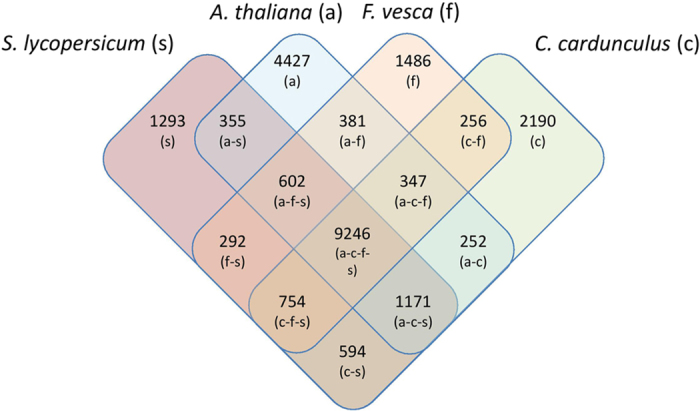
Venn diagram of orthologous gene clusters among Arabidopsis thaliana (**a**), *Cynara cardunculus* (**c**), *Fragaria vesca* (**f**) and *Solanum lycopersicum* (**s**), showing a total of 9,246 common gene clusters.

Comparative analysis of globe artichoke proteins against *Arabidopsis thaliana*, turnip (*Brassica rapa*), strawberry (*Fragaria vesca*), tomato (*Solanum lycopersicum*) and lettuce (*Lactuca sativa*) resulted in 25,981 clusters, which included 153,126 proteins out of 201,990 proteins across the six taxa. Of the 27,196 globe artichoke proteins, 20,509 were grouped into 14,810 gene families, of which 418 are unique families, and 6,687 are singletons. A core set of 6,005 families was shared by the six species of which 2,911 were detected as single copy in all the species (Supplementary Table S6A). The largest number of gene clusters was shared between globe artichoke and tomato, presumably due to their closer evolutionary relationship (*vs*. *Arabidopsis*/strawberry, Fig. 4). The shared gene clusters represent 79.4% (for globe artichoke) and 82.2% (for tomato) of their individual total gene clusters.

We searched for gene families in which a particular species contained a higher number of genes than expected and 46 returned a significant deviation, mostly driven by expansions in either *Arabidopsis*, tomato, strawberry or lettuce. Four clusters, in which globe artichoke had an expanded number of genes, are reported in Supplementary Table S6B. These are annotated as (i) Cystatin-like proteins with the cysteine proteases inhibitor signature (33 members), (ii) replication factor A, C-terminal domain (15 members), (iii) glycosyltransferase family 10 (fucosyltransferase, 8 members); none of these three groups of genes were physically clustered. A fourth group (27 members), containing genes encoding p450 class E enzyme group I proteins was physically clustered in chromosome 13 (15 out of 27 genes, Supplementary Fig. S3A), in a region spanning 800 Kb interspersed with 44 other genes. The phylogenetic analysis suggested that the 15 genes resulted from serial duplication events (Supplementary Fig. S3B).

Five other clusters were over-represented in both globe artichoke and lettuce (Supplementary Table S6B). The first two encoded NB-LRR disease resistance related proteins (with 20 and 19 members), one of which was clustered on chromosome 10 in two separate regions of 2 Mb each (Supplementary Fig. S3A). A third cluster encoded bulb-type mannose specific lectin associated protein kinases (19 members) that localized on three chromosomes (chr. 5, 5 members; chr. 6, 5 members and chr. 9, 4 members). A fourth group encoded pentatricopeptide proteins (36 members). The last group encoded an Allergen Related Protein family (13 members) that was clustered on chromosome 15 over a 1 Mb region. A total of 418 clusters (1,170 genes) were unique to globe artichoke, across the panel of six analyzed species. A GO enrichment analysis showed an over-representation in some genes, particularly in relation to “response to stress”, “phenylpropanoid biosynthesis”, and “lipid biosynthesis” (Supplementary Fig. 3C). The specific genes are widely distributed over the globe artichoke chromosomes; 18 regions had clusters of genes involved in the same biological process (Supplementary Table 6c; Supplementary Fig. S3C). In particular, there are clusters for “Defense Response” on chromosomes 1 (39 loci), 10 (5 loci), 13 (19 loci) and 16 (4 loci), “Auxin-activated signaling pathway” on chromosomes 14 (3 loci), 15 (6 loci), 17 (4 loci) and “Regulation of transcription” on chromosomes 2 (6 loci) and 14 (3 loci).

### Repetitive content of the genome

**Figure 5:**
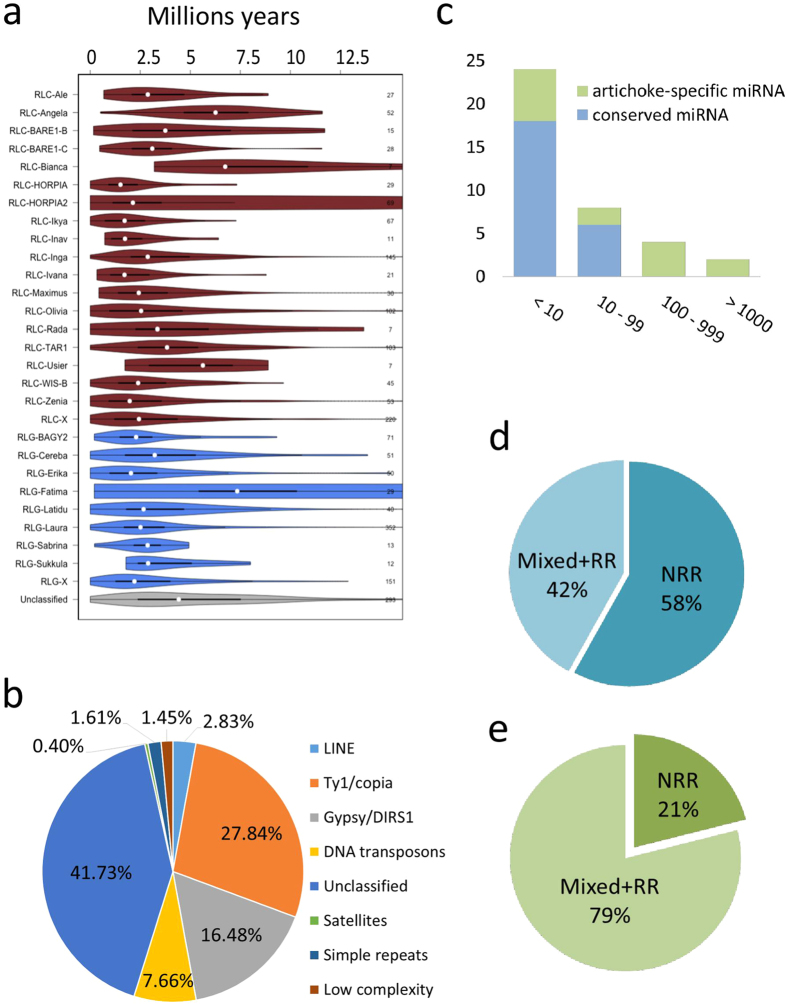
Repeat identification and dating in the genome of *C. cardunculus* and miRNA analysis. **(a)** Overall distribution of the repetitive fraction. **(b)** Distributions of insertion ages in different families of LTR elements. **(c)** Number of loci per miRNA considered as four frequency classes: (i) <10 loci; (ii) 10 to 99 loci; (iii) 100 to 999 loci; (iv) >1000 loci. **(d)** Distribution of Mixed, RR and NRR classes of miRNA within conserved miRNAs. **(e)** Distribution of Mixed, RR and NRR classes of miRNA within globe artichoke-specific miRNAs. RR = miRNAs represented exclusively by loci covered by repeat elements; NRR = miRNAs without any locus covered by repeat elements; Mixed = miRNAs which include both loci covered and not covered by repeats. Conserved miRNAs are present in at least two of the 11 tested species.

Approximately 424 Mb (58.4%) of the assembly was repetitive (Fig. 5a), of which 41.73% could not be classified. The most abundant super-families were class *Ty/1 Copia* (27.84%) and *Gypsy* elements (16.48%) followed by class II DNA elements (7.66%). 14,449 complete LTR retrotransposon-like elements were predicted; of these, 13,080 clustered into 910 families, which were then reduced to 610 by means of 2,142 unambiguously clustered LTR elements. Subsequently, 354 clusters were successfully classified into families and sub-families, while 154 were assigned to main families (RLG - Retrotransposon LTR Gypsy, RLC - Retrotransposon LTR Copia, and RIX - Retrotransposon Unclassified). The 12,107 unclustered elements were aligned with BLAST against TREP database (http://wheat.pw.usda.gov/ggpages/Repeats/) resulting in 7,909 type/family assignments. Of all the annotated repetitive elements, 57% and 37% belonged to the *Gypsy* and *Copia* families, respectively. Insertion dates were calculated based on sequence divergence of the LTRs for all the clustered elements; an expansion was evident at 2.5 Mya (Supplementary Fig. 7). Lower variability in the ages among the *Gypsy* elements was observed, with a tendency to be more stable across time, with the exception of the *Fatima* sub-family. Higher variability was detected between different subtypes of *Copia* elements, with certain sub-families showing a decrease in insertion events over time, while others had a higher proportion of young elements (Fig. 5b).

### Satellite and microsatellite sequences

The inspection of the available assembly for the presence of satellite sequences allowed the identification of several monomers scattered across the genome. The most abundant where three monomers of 96 bp, 94 bp and 103 bp, found 493, 477 and 191 times, respectively (Supplementary Table S7). Other satellite sequences have been identified with minor frequencies. The distribution of satellite motifs across the chromosomes showed a contextual enrichment within presumptive centromeres as suggested by density of repeats (Supplementary Fig. S8). It was not possible to determine a single satellite motif involved in centromeres.

The 17 pseudomolecules and unmapped scaffolds were used for the bulk mining of SSR markers and for the construction of the first microsatellite marker database *CyMSatDB* (*Cynara cardunculus* MicroSatellite DataBase), available at http://www.artichokegenome.unito.it. A total of about 177,200 perfect SSR motifs were identified, including about 27,280 compound SSRs. The SSR loci (namely *CyMSat*) identified were classified on the basis of the repeat motif and their distribution over the pseudomolecules (Supplementary Table S8) as well as the number of repeat units^38^. Di-nucleotides are the most frequent (73.0%), followed by tri- (11.2%), tetra-nucleotides (6.1%) and mono- (4.7%); penta- and hexa-nucleotides are rare (2.4 and 2.5% respectively). Nearly 244,000 imperfect SSR motifs were identified.

### miRNA analysis

Based on the sequence similarity to known miRNAs of 11 species with diverse evolutionary relationships to globe artichoke, 73 different miRNAs (Supplementary Table S9) were computationally predicted in the globe artichoke genome. Since their identification was driven by similarity to known miRNAs, the size of the globe artichoke miRNome was likely underestimated^39^, but sufficient to provide an overview of its basic genomic organization. Although some previously known, conserved miRNAs, such as miR162, and miR482, were not identified, possibly due to their loss or, more likely, to genomic loci missing in the assembly, candidate genes for all but one of the 19 conserved miRNAs reported in previous smallRNA–seq studies of leaves and roots of globe artichoke^40^ were detected. Fifty-eight miRNAs were represented by less than 10 loci, nine numbered between 10 and 99 loci and four between 100 and 999, while two had greater than 1,000 loci (Fig. 5c, and Supplementary file S2). All the miRNAs with more than 100 loci were *C. cardunculus*-specific (referring to miRNAs present in mirBase release 21), although some low-copy species-specific miRNAs were also detected.

The 73 miRNAs of globe artichoke were grouped according to their relationship with repeated sequences in the genome (mainly SSRs, low complexity regions and TEs): (i) miRNAs represented exclusively by loci covered by repeat elements (RR-miRNAs), (ii) miRNAs without any locus covered by repeat elements (NRR-miRNAs), and (iii) miRNAs which included both loci covered and not-covered by repeats (Mixed-miRNAs) (Supplementary Table S10). Interestingly, the majority of miRNAs (~58%, Fig. 5d) present, besides *C. cardunculus*, in at least two of the 11 tested species belonged to the NRR group, while only ~21% of *C. cardunculus*-specific miRNAs felt in this group (Fig. 5e).

Analysis of putative miRNA-targets among the transcripts identified here was carried out on both conserved and putative non-conserved miRNAs using mature sequences present in miRBase, release 21 as a reference. In total, 297 target sites for 57 miRNAs were located in 256 transcripts (Supplementary file S3a). Both conserved and non-conserved microRNAs target multiple genes: as examples, miR396, miR156 and miR395 target 39, 26 and 23 genes, respectively and, among non-conserved miRNAs, miR6108, miR6111 and miR6114 target 28, 24 and 15 genes respectively. A GO enrichment analysis identified several categories that were enriched for miRNA-target transcripts (Supplementary file S3b, Supplementary Fig. S3D). For processes (P), enrichments were observed in: regulation of transcription (GO:0006355, GO:0045449), protein import/transport (GO:0017038, GO:0015031), protein localization in mitochondrion (GO:0070585), protein import/localization in nucleus (GO:0034504, GO:0006606). With respect to functions (F), enrichments were observed in: nucleic acid binding (GO:0003676), protein transporter activity (GO:0008565), and ATP binding (GO:0005524). For components (C) enrichments were present for: nucleus (GO:0005634) and mitochondrial membrane outer membrane translocase complex (GO:0005742).

Many conserved miRNAs were predicted to target known transcription factors related to plant development, morphology and flowering time. Examples include miR160 and ARF (Auxin Response Factor), miR156 and SQUAMOSA promoter binding-like proteins, miR164 and NAC-like proteins, miR172 and AP2-like proteins and miR171 and GRAS-like proteins. Putative artichoke-specific miRNAs mainly target genes associated with enzymes and physiological proteins involved in adaptation such as metal ion-binding proteins involved in ion transport in plasma membrane (miR6108), serine/threonine protein kinases (miR6111), metal ion binding proteins and ATP binding proteins (miR6114), nucleotide binding (miR6110), ATP binding proteins and Leucine Rich Repeat (LRR) as well as protein with serine/threonine kinase activity (miR6118).

### Age of speciation

**Figure 6:**
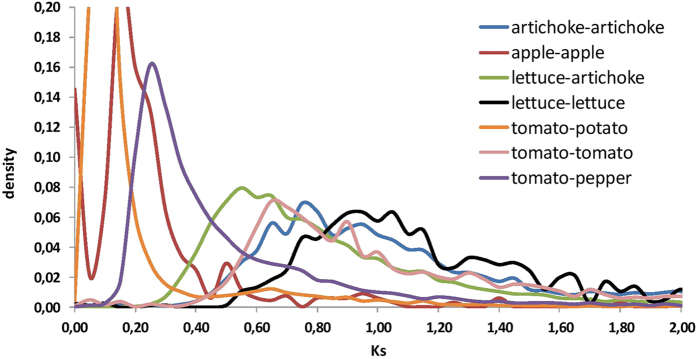
Distribution of synonymous substitutions across paralogs and orthologs as defined through conservation of syntenic blocks. Lettuce and globe artichoke speciation is described with a Ks peak of ~0.55, while ancestral common genome duplication predating the Euasterids II radiation is described by a Ks ~ 0.78 for globe artichoke and ~0.87 for lettuce prior to heterogeneity rate correction, confirming that no WGD occurred after speciation of *Chicoriae* and *Cardueae*.

Distributions of synonymous nucleotide substitutions (Ks) were analysed to investigate ages of speciation (Fig. 6). The shared whole genome duplication(s) at the base of the Compositae family were placed at Ks ~ 0.78 in *C. cardunculus* and Ks ~ 0.95 in *L. sativa*. However, by applying the Ks correction to compensate for nuclear rate heterogeneity across Compositae as estimated elsewhere^19^, the *C. cardunculus* and *L. sativa* WGD were estimated at Ks = 0.84 and Ks = 0.87, respectively, thus providing further evidence for a common Compositae WGD. Divergence of *Cardueae* and *Cichorieae* was calculated at Ks ~ 0.55 and compared with that obtained for pepper and tomato (Ks = 0.27) with the same analysis pipeline, consistent with the previous similar estimation (Ks = 0.30)^24^. Assuming the divergence of pepper and tomato at 19.1 Mya, this places the time of divergence of the *Cardueae*, *Cichorieae* and *Heliatheae* at 35 to 40 Mya.

## Discussion

Here we report the first published genome sequence of a Compositae crop species and make these new genomic resources fully available to the scientific community. The Compositae is the largest family of flowering plants and its ecological success has served as a stimulus for studies of the mechanisms driving speciation and adaptation in plants^19^,^41^,^42^. Our data will enable genome-scale analyses of evolutionary processes among crops, weeds, and wild species both within and beyond the Compositae and will facilitate research and crop improvement in this large and economically important family.

The construction of a reference genome for a highly heterozygous, outbreeding species like globe artichoke is more challenging than for homozygous, inbreeding species. The analysis of a nearly homozygous genotype can facilitate the assembly of a single representative sequence with de Brujin graph-based algorithms. High density genetic analysis is an efficient strategy to validate assemblies and assign contigs and scaffolds to genetic bins ordered along chromosomal linkage groups. Next generation sequencing offers several methods to collect massive amounts of genotyping data in a cost-effective manner. Some rely on complexity reduction with restriction enzymes (GBS^43^, RADseq^44^, ddRAD^45^, MSG^46^) that are robust tools for NGS-based genotyping. However, the choice of restriction enzymes and the rate of polymorphisms between the parental genotypes can define the numbers and distribution of informative loci that cover only a subset of sequences. Several approaches have been described that utilize low coverage whole genome sequencing of segregating individuals^28^,^29^. However, they require some *a priori* knowledge of at least one parental haplotype along with the expectation of homozygous parental lines. Moreover, assignment of genotypes from shallow sequencing data (<1×) is only robust when two known alternative haplotypes are expected to segregate as homozygotes, thus restricting their application to RILs. Other populations, such as F_2_ and BC progeny, require much higher sequencing coverage to permit confident calls of heterozygous genotypes. Situations in which both parents are highly heterozygous and their F_1_ progeny is segregating in a two-way pseudo-testcross pattern, render previously described methods unsuitable for low coverage whole genome sequencing data.

In order to provide the draft sequence of chromosomal pseudomolecules of the highly heterozygous, outbreeding *C. cardunculus*, we developed the novel SOILoCo methodology, which employs low coverage genotyping-by-sequencing of an F_1_ progeny based on haplotype phase information computed at parental level. The method does not require *a priori* reconstructed parental haplotypes and can be applied regardless of which, if any, haplotype is present in reference genome sequence. Our pipeline proved successful in positioning and orienting scaffolds of the *de novo* reference assembly of globe artichoke. The accuracy of our approach was validated by aligning the two independently generated parental maps. Although the pipeline was developed to analyse F_1_ progeny data, it also could be utilized for mapping with other progenies, such as F_2_, RILs, and BC populations.

The first reference genome for *C. cardunculus* provided the possibility to analyse both its coding gene space and non-coding elements. Expansion of multigene families through gene duplication is an important component in the evolution of new gene functions^47^. Moreover, it has become evident that some of the complexity of higher organisms, which is related to an increase in the number and complexity of regulatory pathways derives from an increase of the size of non-coding regions^48^. Repetitive elements, particularly transposable elements, which are one of the major components of most plant genomes, have largely contributed the formation and family expansion of miRNAs, a major class of small non-coding RNA^49^,^50^.

Analysis of the globe artichoke gene-space revealed specific expansions of some gene families such as the genes encoding phytocystatins (PhyCys), which have been reported to be highly effective in pest defense^51^, and P450 proteins which are potentially involved in production of secondary metabolites. Some other expansions of gene clusters encoding NBS-LRR proteins were also shown to be present in lettuce. All these genes are candidates for further investigation to understand their role in biotic stress resistance and generating the repertoire of secondary metabolite in globe artichoke. Physically clustered and/or species-specific genes involved in the same biological process are a frequent trait in globe artichoke. However, comparative genomic analysis through the whole genome re-sequencing of *C. cardunculus* wild relatives will be necessary to determine all the specific genes underlying the globe artichoke’s unique phenotype.

Although many studies concerning miRNA loci have focused on the relationship between miRNAs and TEs, the study of SSRs in pre-miRNAs is becoming an interesting topic^48^,^49^,^50^. Several putative miRNA-SSR have been found throughout the genome of globe artichoke; di-nucleotides (mainly TA/AT in miRNA-SSR) are the most frequent (88%), followed by tri- (5.8%) and tetra-nucleotides (2.7%) while mono, penta and hexa-nucleotides are rarest (0.1, 1.6 and 1.7% respectively) similar to what has been reported in rice^51^. Besides the possible application of microsatellites as genetic markers for molecular breeding with miRNA genes^52^, the SSR variation (expansion or contraction) might affect the miRNA products and cause significant changes in their activity. Future studies will be devoted to the analysis of the potential of polymorphic miRNA-SSR.

Many conserved miRNAs detected in the globe artichoke genome are predicted to target well known biological processes confirming, on the basis of a more comprehensive data set, previous reports^39^. The findings on non-conserved artichoke miRNAs are also of interest because of recent work in other species suggesting that such miRNAs may contribute to stress responses (Qin *et al.*, 2014). Unlike ancient, conserved miRNAs, which are often highly expressed, young (lineage-specific or species-specific) miRNAs tend to be weakly expressed, processed imprecisely, and lack functional targets, implying that they are non-functional. However, a handful of newly derived miRNAs display high levels of expression in specific tissues or under particular stress treatments, consistent with a possible role in environmental adaptation^53^. Future analysis based on smallRNA-seq, degradome sequencing and/or 5′ RACE are required to test this hypothesis and to reveal details of the potential biological role(s) of non-conserved miRNAs in stress responses of globe artichoke.

Analysis of LTR insertion times suggests that most of the genome expansion of *C. cardunculus* was relatively recent and mainly occurred after divergence from the Heliantheae - Cichorieae lineage. Recent increase in retrotransposon activity has also been reported in another Compositae species, sunflower^54^. This pattern could be related to evolutionary events rather than a direct relationship between the expansions since they occurred after the separation of the tribes. The sunflower expansion is more recent, less than 1 Mya, than the expansion that occurred 2.5 Mya in the globe artichoke lineage. This could be related to the second WGD event in the Heliantheae lineage and also explain part of the difference in genome size between the two species.

In summary, the genomic sequences and features revealed in the present study not only enhance the taxonomic breadth of the data available for comparative plant genomics, but will also facilitate the identification of evolutionarily interesting and economically important genes from related species within the Compositae family.

## Methods

### Genome Sequencing and Assembly

A globe artichoke Brazilian breeding line, designated ‘2C’, was subjected to three cycles of selfing to significantly reduce the level of heterozygosity and to be used as the source of DNA for sequencing and *de novo* assembly of the reference genome sequence. High-quality genomic DNA was extracted from leaves of seedlings using a CTAB protocol. Following ethanol precipitation, DNA was recovered with a Pasteur pipette to preserve large DNA molecules (>40 Kb). RNAse A was used to remove RNA contamination. The DNA quality was evaluated with pulsed field gel electrophoresis (PFGE) on a CHEF-DRIII system (BioRad, Hercules, CA, USA). The level of residual heterozygosity was assessed by analysing 115 SSR markers that we had previously developed^30^,^31^.

To prepare overlapping paired-end libraries, genomic DNA was fragmented by sonication using a Covaris S220 (Duty Cycle: 10%, Intensity: 5, Cycle per Burst: 200, Time: 360 s; Covaris, Woburn, MA). End-repair and A-tailing procedures followed standard Illumina protocols. PCR-free barcoded adapters (Biooscientific, Austin, TX, USA) were ligated to the fragments following the standard Illumina TruSeq protocol. Fragments of 280–320 bp were selected using PippinPrep instrument (Sage Science, Beverly, MA, USA) and extracted with QIAquick columns (Qiagen, Inc., Venlo, Netherlands). Library preparation followed the Illumina TruSeq protocol, with minor modifications (i.e. five enrichment PCR cycles). Quality and quantity of the libraries were assessed with a Bioanalyzer 2100 instrument (Agilent, Inc., Santa Clara, CA, USA) and qPCR, respectively. Sequencing was performed with two Illumina HiSeq2000 lanes.

To obtain mate-pair libraries, DNA from genotype ‘2C’ was fragmented using a Hydroshear instrument (Digilab., Marlborough, MA, USA) using manufacturer suggested settings for 1.4 to 8 Kb (medium assembly, 20 cycles) with speed set to 14 to target 5 to 8 Kb fragments and speed set to 10 for the 3 to 4 Kb range. DNA fragments were then separated by PFGE running under “Restriction fragment” conditions as suggested in the manual. Fragments of 3 to 4 Kb and 6 to 7 Kb were excised from the gel and extracted with Zymoclean Large fragment DNA Recovery kit (Zymo Corp., Irvine, CA, USA.). Mate-pair libraries were generated following the Illumina Mate-pair V2 TruSeq protocol except for the use of barcoded Biooscientific PCR-free adapters in the ligation step. Quality and quantity were assessed as described above. Sequencing was performed with two Illumina HiSeq2000 lanes for the 3 Kb mate-pair library and 50% of total molar concentration of one HiSeq2000 lane for the 6 Kb mate-pair library.

A total of 163 individuals of an extant F_1_ mapping population derived from a cross between the globe artichoke genotype ‘Romanesco C3’ and the cultivated cardoon genotype ‘Altilis 41’^14^, were sequenced at low coverage and used for anchoring the scaffolds to a genetic map. DNA from parental and offspring genotypes was extracted using a CTAB protocol^55^. Genomic DNA of each parent/individual was fragmented with a Bioruptor instrument (Diagenode, Liège, Belgium) using 15”/90” on/off time for 9 cycles. End-repair and A-tailing procedures followed the standard Illumina protocol, except PCR-free barcoded adapters (Biooscientific) were used during the ligation step. Libraries were quantified using an Analyst plate reader and diluted to a concentration of 5 nM. Five pools (1 to 5) were constituted by aggregating 48, 46, 23, 23 and 23 samples respectively and sequenced on an Illumina HiSeq2000. Pools 1 and 2 were sequenced using two lanes each, while pools 3, 4 and 5 were sequenced using one lane each (Supplementary Fig. S4). Libraries of the parental genotypes were sequenced using one HiSeq2000 lane each. Additional libraries with an insert size of 600 to 700 bp were produced for each parent and sequenced in one lane.

Illumina reads for both paired-end and mate-pair libraries were processed to remove adapter contaminants and low quality bases using the “Barcode tools” script suite (http://comailab.genomecenter.ucdavis.edu/index.php/Barcoded_data_preparation_tools). High quality reads were assembled using the ALLPATHS-LG software^32^, with default parameters and the “HAPLOIDIFY = True” option. Quality metrics for the assembled genome were calculated using Assemblathon_stats.pl (http://korflab.ucdavis.edu/).

### Gene prediction and annotation

Repeat elements were identified *de novo* with the RepeatModeler pipeline and masked with RepeatMasker software. Perfect, imperfect and compound SSRs were identified using the SciRoKo SSR-search module (http://kofler.or.at/bioinformatics/SciRoKo). Satellite motifs were discovered using TRF^56^ and filtered for a minimum length of 80 bp and and 4 repeated monomers. Sequences were then clustered at 90% identity using CD-HIT^57^ to retrieve representative monomers; only clusters with at least 5 elements were retained. Occurrences on the assembly were obtained via BLASTn alignment of representative sequences with a minimum identity of 90%; only independent non-consecutive matches with a minimum distance of 50 bp were considered for statistics.

Gene prediction utilized reiterative runs of the MAKER suite^58^. Both EST sequences and RNAseq data were used to guide gene annotation. RNAseq data of eight globe artichoke and cardoon genotypes was retrieved from SRA archive (PRJNA72327). EST sequences for *C. cardunculus* and other available Compositae species were downloaded from the NCBI as well as the non-redundant (nr) protein database for *Viridiplantae*. RNAseq reads were aligned to the reference assembly using TopHat2 aligner and *de novo* transcripts were assembled using the Cufflinks package with default parameters. A first run of MAKER annotation pipeline was carried out by employing only transcript assemblies along with ESTs and protein alignments to retrieve candidate gene predictions. After filtering for high quality preliminary predictions, HMM models for Augustus and SNAP *ab initio* gene prediction algorithms were produced. Then, by utilizing these *ad-hoc* HMM models, along with EST and proteins alignments as supporting evidence, final gene models were obtained in a second run of MAKER.

Predicted protein sequences were functionally annotated using InterproScan5^59^ against all the available databases (ProDom-2006.1, Panther-7.2, SMART-6.2, PrositeProfiles-20.89, TIGRFAM-12.0, PrositePatterns-20.89, PfamA-26.0, SuperFamily-1.75, PRINTS-42.0, Gene3d-3.5.0, PIRSF-2.83, HAMAP-201207.4, Coils-2.2). In parallel, the same protein set was clustered using OrthoMCL^60^ with default parameters. Putative functions were assigned to each protein cluster based in the InterproScan functional predictions for all the members in the cluster. Protein datasets for *Arabidposis thaliana*, *Brassica rapa*, *Fragaria vesca* and *Solanum lycopersicum* (September 2014), were download from Phytozome V9^61^. A predicted proteome was used for *Lactuca sativa* (unpublished data). All the proteins were clustered with the *C. cardunculus* using OrthoMCL to generate orthologous clusters with default parameters.

OrthoMCL Clusters, with species having expanded number of genes, were mined using a chi-square test comparing the counts of genes/species against an expected value. Only clusters with a mean number of genes above five were considered. These clusters were analysed for a significant deviation from mean gene count for species adopting a Bonferroni corrected p-value (p < 0.05). GO enrichment in the globe artichoke specific clusters was calculated with AgriGo (http://bioinfo.cau.edu.cn/agriGO) and visualized with the REVIGO suite (http://revigo.irb.hr). Genomic regions carrying cluster of genes were highlighted by aligning genomic full-length genes on the reference genome using BWA (bwa-sw algorithm; http://bio-bwa.sourceforge.net).

### miRNA analysis

Candidate miRNA-coding sequences for each chromosomal pseudomolecule (1 to 17) were identified using MIReNA software^62^ from known miRNAs sequences (miRBase release 20) from a diverse array of plants and algae: *Helianthus tuberosus, Solanum lycopersicum, Solanum tuberosum, Nicotiana tabacum, Vitis vinifera, Arabidopsis thaliana, Oryza sativa, Brachypodium distachyon, Picea abies, Physcomitrella patens, Chlamydomonas reinhardtii.* MIReNA software has already been proven to successfully predict pre-miRNAs in plants^63^. MIReNA was run with default parameters except for the maximum number of admitted errors between known miRNAs and putative miRNAs, which was set to 10 (the value is given in percentage).

All miRNA predictions were screened for transcriptional evidence on the basis of RNA-seq data generated in previous studies^11^, re-analysed using HTSeq-count^64^. The predicted miRNA genomic coordinates were adopted as genomic features counting how many reads mapped to each feature in overlap resolution mode “intersection-strict”. Counts of more than 10 mapped reads/miRNA genomic loci were considered to represent evidence of expression. Putative miRNA-coding sequences were BLASTed (BLASTn, locally installed, default parameters) against *C. cardunculus* ESTs downloaded from NCBI dbEST (release 130101) and *C. cardunculus* transcript sequences^11^. BLASTn results were filtered to retain only those sequences with a nucleotide identity greater than or equal to 90% compared to the aligned sequence and with an alignment length equal or greater than 30 nt to limit the false positives arising from possible miRNA targets^65^. All pseudomolecule sequence assemblies were analyzed with respect to repetitive elements: the coordinates (start and end position) of predicted miRNA-coding sequences were superimposed with the coordinates of the identified repetitive segments using Bedtools intersect (default parameters with -loj option) and those with at least one nucleotide overlap were labeled as repeat-related regions.

For analysis of miRNA targets, transcribed sequences were subjected to psRNATarget (http://plantgrn.noble.org/psRNATarget) analysis against the mature *C. cardunculus* miRNA retrieved from miRBase (Release 21). A maximum expectation of 2.5 was adopted, allowing a maximum energy to unpair the target site of 25, and considering 17 bp upstream and 13 bp downstream of the target site sequence. Inhibition of translation was considered for mismatches in the 9th to 11th mature miRNA nucleotides. Any enrichment of GO terms was verified by comparing the putative miRNA targets against the whole genome GO annotation dataset of globe artichoke by means of the AgriGo suite collecting terms with P values <e^−5^ and false discovery rate <0.01. The DAVID suite (https://david.ncifcrf.gov/) was used to functionally cluster miRNA targets recognised by miRNA sequences (by family).

### Genome anchoring

A novel pipeline that we named SOILoCo (Scaffold Ordering by Imputation with Low Coverage) was developed to impute haplotypes from low-coverage sequencing data of F_1_ progeny from a cross between heterozygous, phase-unknown parents. The core part of the SOILoCo pipeline is written in Perl and available at https://bitbucket.org/dscaglione/soiloco with a subsample of the dataset used in this work. Primary input data was generated as follows: Each set of parental reads was aligned to the draft reference assembly using the BWA aln algorithm with default parameters. SNP calling was performed with samtools mpileup on each parent separately and both together in a multi-sample call. To populate the initial SNP table, a minimum mapping quality of 25 and SNP quality of 20 were required. Similarly, each progeny read set was aligned and a multi-sample call was carried out on all 163 samples. HAPCUT algorithm (v0.5^36^,) was used to reconstruct haplotype blocks of each parent utilizing its individual SNP call table, 100 iterations were performed. The first script of the pipeline (“TC-separator.pl”) leverages two-sample parental SNP calling and the phase assembly of each to (i) split candidate testcross segregating SNP sites for each parent, (ii) divide coordinates as segregating from either phase-0 or phase-1 (type) and return an updated version of the progeny-wide SNP calling table (in VCF format), with scaffolds organized in haplotype blocks and types (depending on the phase of the segregating allele). Figure 1 shows how SNPs within a haplotype block of parent P1 are sorted depending whether they segregate from phase-0 or phase-1 with respect to the reference genome sequence. An ancillary script (“vcf2string.pl”) was then used to parse parent-specific VCF files and construct the sequence of calls for each individual over a given haplotype block; at this stage population-wide allele frequency constraints were applied to filter out possible false testcross SNPs (TC-SNPs). For this study, given the expected haplotype segregation (1:3), minor allele frequencies (MAFs) in the range of 0.075 to 0.425 were allowed. The Markov chain–based algorithm embed in “gt-hmm.pl” applies a Viterbi decoding procedure given matrices of emission and transition probabilities of unknown genotypes in the finite states diagram depicted in Supplementary Fig. S5. Probability distributions were given by *T*, the genotype transition probability, *E*_*het*_ the false heterozygous miscalling probability, E_hom_ the false homozygous miscalling probability, and *C* the probability of heterozygous call by multiple read mapping (a factor given by sequencing depth, assumed to rely on Poisson distribution). A dynamic programming algorithm was used to calculate most likely genotype paths of the haplotype block for the segregating progeny individual. Following the Viterbi forward decoding, given X = {X_1_, X_2_, …, X_n_} the sequence of observed genotype calls and Z = {Z_1_, Z_2_, …, Z_n_} the unknown path of underlying genotype states, the most likely path of states Z*, originates from


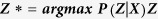


by finding the maximized probability of


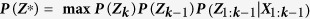


we used P(Z^2^) as the second highest probability, to consider candidate alternative paths. We applied a minimum LOD score to accept genotype imputations, where


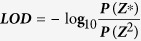


Thus, whether an alternative path is decoded, with a probability (as LOD score) lower than its minimum threshold, the best genotype call is rejected due to ambiguity. In case a candidate best path contained one or more state transitions (i.e. genotype switching), trimming routines are invoked to remove imputation errors. Such procedures consider relative length of two juxtaposed genotype sequences and their relative SNP density. Longer paths and SNP dense regions are favoured in contrast to short paths and relatively SNP poor regions, given maximum ratios of length and SNP density. The analysis was run with probabilities set as follows: T = 10e^−8^, C = 0.2, E_hom_ = 0.01, E_het_ = 0.01. The minimum length of an observed genotype call was set to 6. Maximum accepted ratios of SNP density and path length were both set to 0.2. Minimum LOD to accept the best call path was 2. If any of these conditions was not satisfied, the genotype imputation for that particular individual was not generated. Afterwards, “TC_string_merger.pl” was used to join (where necessary) genotype imputation by complementary sets of SNPs over a haplotype block given the segregation from each homologous chromosome (Fig. 1). This procedure was implemented in order to recover missing imputations and to correct wrong low-quality ones. A maximum ratio of missing genotypes per block/type was set to 0.30, while a minimum consensus of 0.50 between each segregation type was required to initiate consensus calling. In the case of contrasting genotype calls between types, the imputation with the highest LOD score was retained. Finally, “calls2csvr.pl” was used to convert the type-merged genotype calls to a segregation data matrix (in a format accepted by R/qtl) by filtering for minimum genotyping ratio of 0.70, and genotype distortion X^2^ p value < 0.03.

Linkage analysis was performed by grouping haplotype-block markers with the “R/qtl” package, with minimum LOD = 10, rec ≤0.20. Ordering was carried out with MSTmap software (http://alumni.cs.ucr.edu/~yonghui/mstmap.html), using the Kosambi function to estimate distance and the maximum-likelihood function to infer correct order. The ordering step was iterated three times, each time correcting genotype calls with the “SMOOTH.pl” script, which is a Perl implementation of the SMOOTH correction algorithm^37^. After each parental map was obtained, scaffold position was calculated by averaging all the genetic positions within a single haplotype block, while correlation of genetic over physical distances with absolute Pearson coefficient > 0.50 was used to infer scaffold orientation. The two maps were finally integrated using the MergeMap algorithm (http://alumni.cs.ucr.edu/~yonghui/mgmap.html) to maximize the ordering and orientation information.

### Repeat elements annotation and dating

Global evaluation of repeats content in the assembly was obtained with RepeatModeler (http://www.repeatmasker.org/RepeatModeler.html), along with the RepBase database for *viridiplantae* (http://www.girinst.org/repbase). LTR elements were characterized and dated following the methods described elsewhere^54^. Genomic sequences were mined for LTR elements using the program ltr_harvest^66^ from the Genome Tools suite to obtain a dataset of predicted LTR elements (with conditions ‘-mintsd 4 -mindistltr 4000 -maxlenltr 4000 -outinner’). We then used ltr_digest to annotate the predicted elements for their main components (PPT, PBS) and the inner protein coding domains. For predictions of the inner protein coding domains, 43 domains were selected from PFam^67^ starting with those employed by Steinbiss^68^. Additional domains were searched using the terms “retrotransposon”, “reverse transcriptase”, “gag transposon”; and also obtaining domains related to the previews results through the PFam ‘domain organization’ tool. Sequence alignments for the Seed dataset and Representative Proteomes at 35% and 75% cut-off were downloaded and HMM’s were generated from them using HMMER2.3.2^69^. For clustering of the predicted elements each of the domains were grouped independently using VMatch^70^ and then LTR’s were grouped using the method used elsewhere^68^. Family classification of all the predicted elements was done as in Wicker *et al.*^71^, using BLASTn and BLASTx searches against the TREP database.

After clustering and annotating the predicted LTR elements into repeat families, the age was calculated as in^72^,^73^ with modifications. A multiple sequence alignment of all the 3′ LTR and 5′ LTR was done using Clustal-Omega 1.2^74^. The alignment was then used to calculate the estimated sequence divergence using BaseML from PAML^75^ based on the similarity between the LTR’s. The estimated divergence between the 3′ LTR and 5′ LTR of each element was used to calculate the age using the formula T = d/2r, with d as the divergence estimate and where r is 1 × 10^−8^ as calculated elsewhere^76^ for host-encoded genes and adjusted for the higher evolution rate of TEs compared with genes.

### Age of speciation

Syntenic genes between artichoke and lettuce were identified using SynMap^77^ on the CoGe platform (http://genomevolution.org) using Last aligner (C-score filter = 0.4) and retrieving syntenic blocks with DAGchainer adopting the following parameters: -D 10 -A 3 -Dm 20. Synonymous substitution densities (Ks) among and between different genomes distribution were calculated over syntenic genes, as described above. Genome annotations as present on the CoGe database were used (*Solanum lycopersicum*: id12289, *Solanum tuberosum*: id16729, *Malus* x *domestica*: id20240, *Capsicum annuum*: id22828).

**URLs:**

- Globe artichoke GBrowse, http://gviewer.gc.ucdavis.edu/cgi-bin/gbrowse/Artichoke_v1_1.

- SOILoCo scripts, https://bitbucket.org/dscaglione/soiloco.

## Additional Information

**Accession codes:** Genome sequence data and annotation have been deposited on NCBI as WGS genome entry and it is currently hosted as validated submission SUB874020 and will be publicly released upon successful peer-review of this manuscript. The version described in this paper is the first version, V1.0. Sequence reads of genome sequencing have been deposited in NCBI sequence read archive (SRA) under project number PRJNA238069. A GBrowse interface to access genome data and its annotation is available at gviewer.gc.ucdavis.edu/cgi-bin/gbrowse/Artichoke_v1_1.

**How to cite this article**: Scaglione, D. *et al.* The genome sequence of the outbreeding globe artichoke constructed *de novo* incorporating a phase-aware low-pass sequencing strategy of F_1_ progeny. *Sci. Rep.* **6**, 19427; doi: 10.1038/srep19427 (2016).

## Supplementary Material

Supplementary Information

Supplementary Dataset S1

Supplementary Dataset S2

Supplementary Dataset S3

## Figures and Tables

**Table 1:** *De novo* sequencing statistics.

Library	# raw pairs of reads	# input filtered pairs	Reads used for assembly (%)	Genome coverage	# pairs in assembly	Physical coverage	Effective insert size
170 bp PE (“A”)	178,243,636	123,227,466	80.3	29.9	97,014,821	26.4	159 +/− 20
170 bp PE (“B”)	222,871,406	213,504,271	83.3	53.6	175,100,448	53.4	182 +/− 20
3 Kb mate pairs (“A”)	188,985,324	163,488,132	49.4	24.2	43,253,631	199.5	2,711 +/− 424
3 Kb mate pairs (“B”)	164,047,981	131,650,064	49.8	19.7	35,198,713	161.6	2,694 +/− 429
6 Kb mate pairs	42,875,964	36,455,448	51.6	5.6	8,232,841	84.5	5,609 +/− 441

“A” and “B” represent separate sequencing libraries. Overall, a total of 133.7 Gb (133×) of sequences were retained for assembly.

**Table 2:** Genome assembly statistics.

Features	Contigs	Scaffolds
Number of sequences	79,681	13,588
Sequences/Mb	121.6	18.9
Total length	654.6 Mb	724.7 Mb
L_50_	17.5 Kb	125.8 Kb
N_50_	10,596	1,411
L_90_	3.4 Kb	28.7 Kb
N_90_	41,711	6,109
Number of sequences >10 Kb	20,561	9,187
Median size of gaps in scaffolds	–	660 +/− 86 bp

**Table 3:** Statistics on predicted genes.

Features	Exons	CDS	Genes	Introns	Transcripts (including isoforms)
Count	154,207	148,979	26,889	121,675	27,121
Average length	246 bp	220 bp	5,450 bp	850 bp	5,422 bp
Median length	141 bp	127 bp	3,669 bp	334 bp	3,656 bp
Total length	37,965,657 bp	32,797,360 bp	146,530,795 bp	103,504,686 bp	147,050,453 bp
Average coding length	–			–	1,215 bp
Median coding length	–			–	996 bp
Total coding length	–			–	32,946,339 bp

**Table 4:** Statistics on the predicted genes for each chromosomal pseudomolecule.

Chr	Gene models	Trascripts	Exons	Avg. exons per gene	# syntenic genes with *L. sativa*	
1	2,630	2,647	15,479	5.89	1,942	(73.8%)
2	2,351	2,370	13,909	5.92	1,639	(69.7%)
3	1,868	1,888	11,068	7.45	1,187	(63.5%)
4	962	972	5,809	6.04	703	(73.1%)
5	1,640	1,653	8,632	5.26	1,036	(63.2%)
6	903	915	5,133	5.68	530	(58.7%)
7	907	914	5,190	5.72	604	(66.6%)
8	1,196	1,209	7,126	5.96	861	(72.0%)
9	1,006	1,012	5,550	5.52	785	(78.0%)
10	1,436	1,444	8,692	6.05	872	(60.7%)
11	1,453	1,462	9,001	6.19	1,238	(85.2%)
12	1,404	1,415	7,814	5.57	898	(64.0%)
13	1,801	1,815	10,157	5.64	1,140	(63.3%)
14	646	651	3,515	5.44	396	(61.3%)
15	1,466	1,478	8,288	5.65	1,024	(69.8%)
16	949	960	5,830	6.14	556	(58.6%)
17	1,277	1,290	7,435	5.82	821	(64.3%)
Unmapped	2,994	3,026	15,576	5.19		0
**Total**	**26,889**	**27,121**	**154,207**	**5.84**	**16,232**	**(67.9%)**
