# Corrigendum: The genome sequence of the outbreeding globe artichoke constructed *de novo* incorporating a phase-aware low-pass sequencing strategy of F_1_ progeny

**DOI:** 10.1038/srep25323

**Published:** 2016-05-23

**Authors:** Davide Scaglione, Sebastian Reyes-Chin-Wo, Alberto Acquadro, Lutz Froenicke, Ezio Portis, Christopher Beitel, Matteo Tirone, Rosario Mauro, Antonino Lo Monaco, Giovanni Mauromicale, Primetta Faccioli, Luigi Cattivelli, Loren Rieseberg, Richard Michelmore, Sergio Lanteri

Scientific Reports 6: Article number: 1942710.1038/srep19427; published online: 01202016; updated: 05232016.

In this Article, there are typographical errors in the legend of Figure 5.

“**(a)** Overall distribution of the repetitive fraction. **(b)** Distributions of insertion ages in different families of LTR elements”.

should read:

“**(b)** Overall distribution of the repetitive fraction. **(a)** Distributions of insertion ages in different families of LTR elements”.

