# Supplementary material for: The genome sequence of the outbreeding globe artichoke constructed de novo incorporating a phase-aware low-pass sequencing strategy of F1 progeny
Source: Sci Rep. 2016 Jan 20;6:19427. doi: 10.1038/srep19427 (PMC4726258; doi:10.1038/srep19427)
Supplement: Supplementary Information [file srep19427-s1.pdf]

The genome sequence of the outbreeding globe artichoke constructed *de novo* incorporating a phase-aware low-pass sequencing strategy of F_1 progeny

Davide Scaglione[1,2,6,*], Sebastian Reyes-Chin-Wo[2], Alberto Acquadro[1,*], Lutz Froenicke[2], Ezio Portis[1], Christopher Beitel[2], Matteo Tirone[1], Rosario Mauro[3], Antonino Lo Monaco[3], Giovanni Mauromicale[3], Primetta Faccioli[4], Luigi Cattivelli[4], Loren Rieseberg[5], Richard Michelmore[2,7], Sergio Lanteri[1,7]

[1] DISAFA, Plant Genetics and Breeding, University of Turin, Grugliasco, Italy
[2] The Genome Center, University of California, Davis, CA, USA
[3] Di3A, University of Catania, Catania, Italy
[4] Consiglio per la ricerca in agricoltura e l'analisi dell'economia agraria, Genomics Research Centre, Fiorenzuola d'Arda, Italy
[5] University of British Columbia, Vancouver, BC, Canada
[6] Current address: IGA Technology Services, Udine, Italy
[7] Joint senior authors.
[*] Corresponding author.

Inventory of Supplementary Information:

- Supplementary Figures S1-S8
- Supplementary Tables S1-S10
- Supplementary data
- Supplementary file list (S1, S2, S3)

Supplementary Figures S1-S8

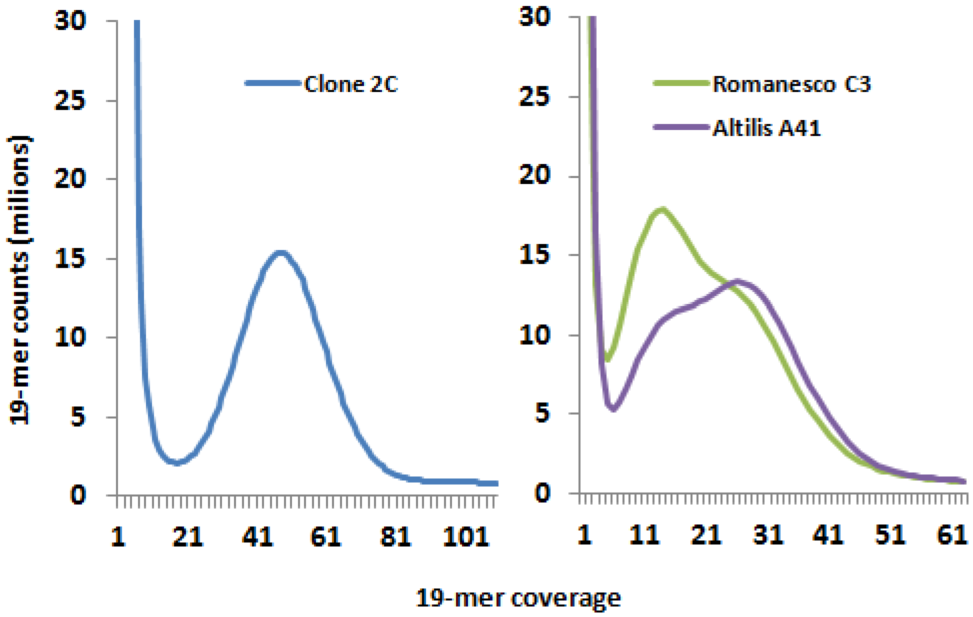

Figure S1 | K-mer spectra analysis using 19-mer counts. Clone "2C" (left) showed significantly lower heterozygosity compared to that of cultivated "Romanesco C3" artichoke and "Altilis A41" cardoon genotypes (right). Among cultivated genotypes the difference between globe artichoke and cardoon was also evident and in agreement with previous reports (Portis *et al.*, 2005).

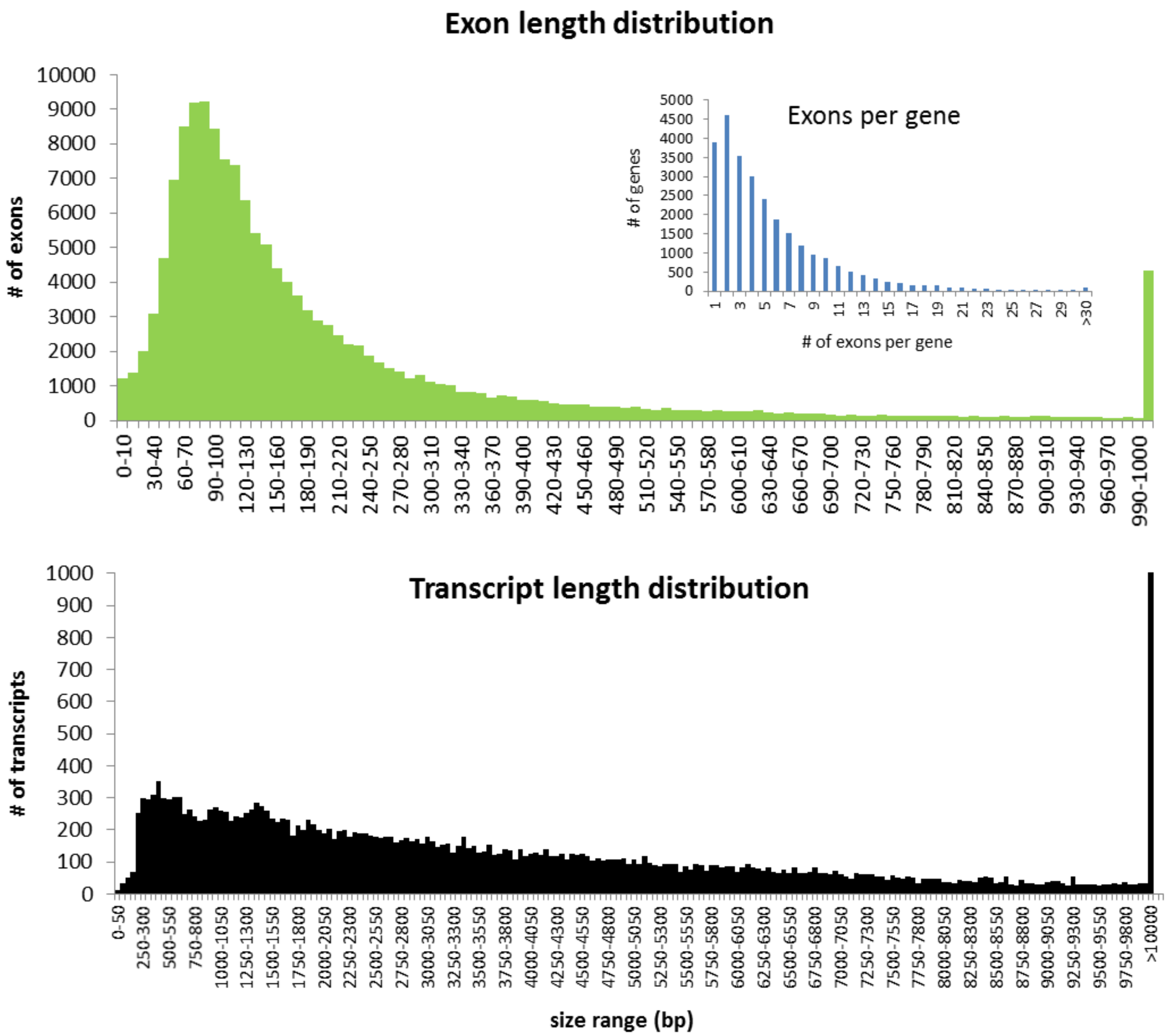

Figure S2 | Exon and transcript length distribution.

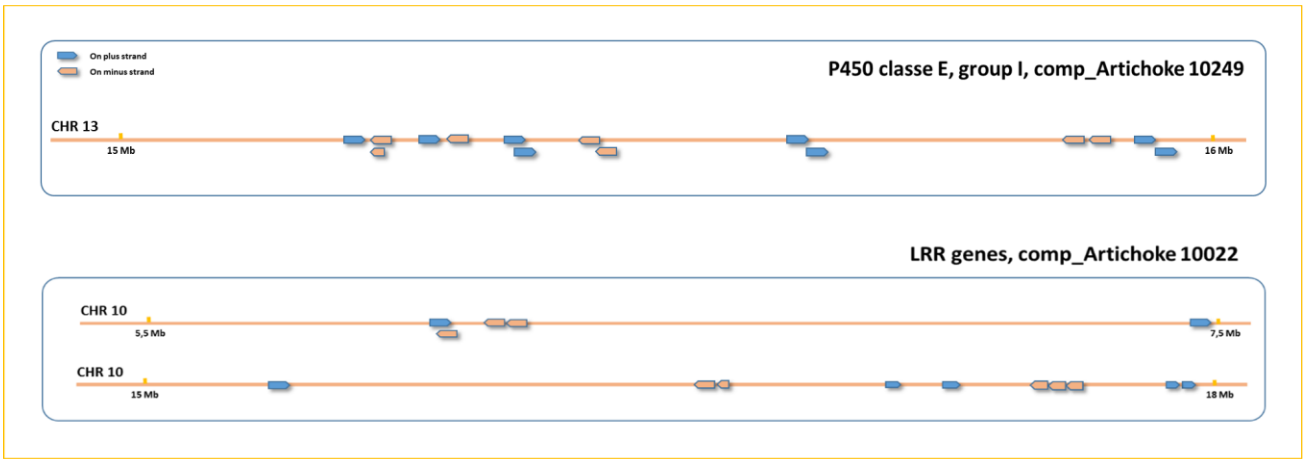

Figure S3A | Gene clustering of some globe artichoke specific OrthoMCL gene groups

Phylogeny of globe artichoke expanded P450 class E, group I, comp_Artichoke 10249

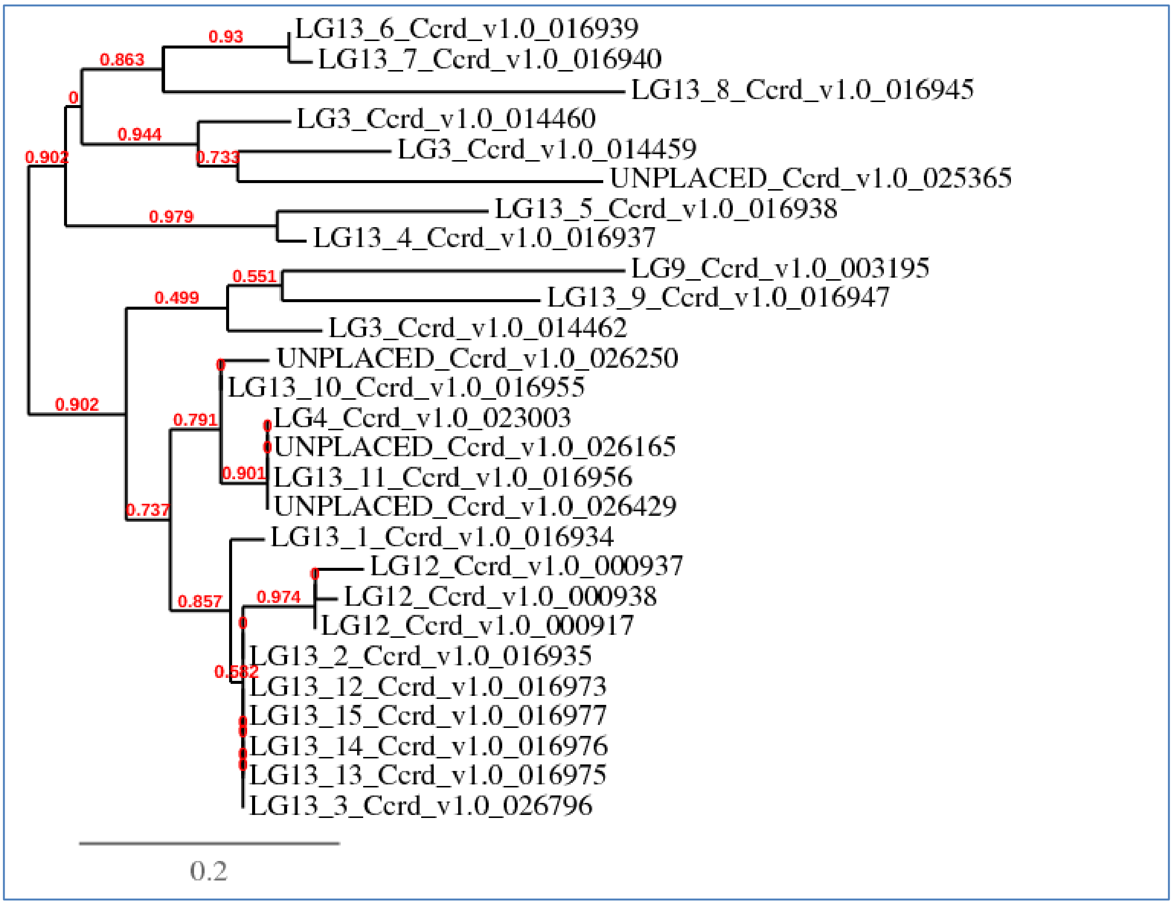

Figure S3B | Phylogeny of globe artichoke expanded P450 class E, group I, comp_Artichoke 10249.

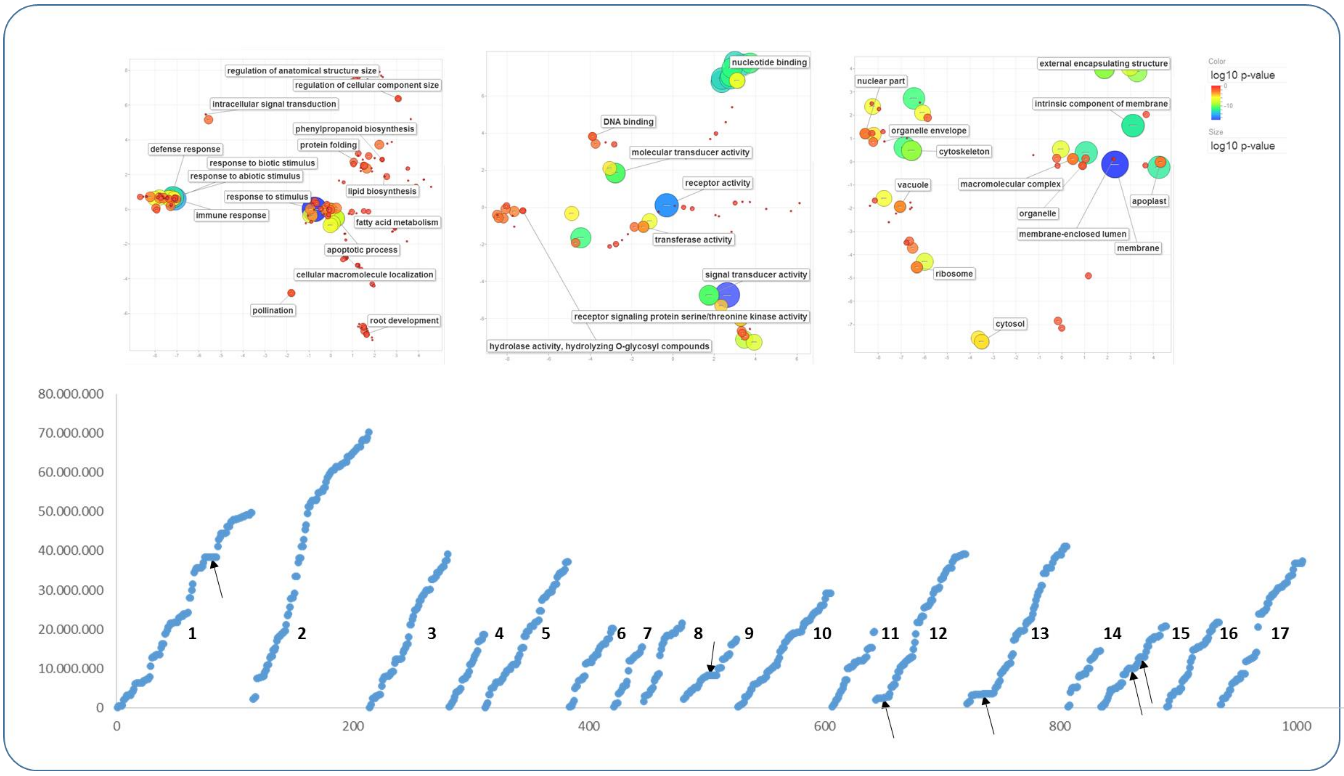

Figure S3C | GO enrichment diagram for the 1170 globe artichoke specific genes in the process, function and component. Enrichment was calculated with AgriGo (http://bioinfo.cau.edu.cn/agriGO) and visualized with the REVIGO suite (http://revigo.irb.hr). Genomic distribution of the 1,170 globe artichoke specific genes along the 17 chromosomes. Black arrows show gene clusters.

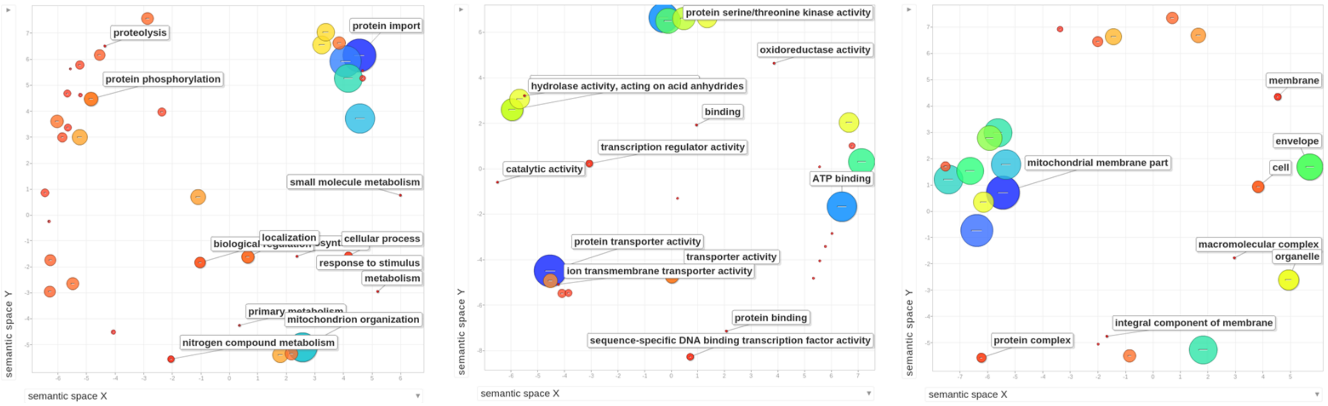

Figure S3D | Analysis of MiRNA target enrichment in the globe artichoke transcriptome. (a) Representation GO category enriched in the miRNA target transcriptome subset separated in the three component (BP, MF, CC from left to right); X axis is expressed in log10 p-value, Y axis is expressed in log10 p-value.

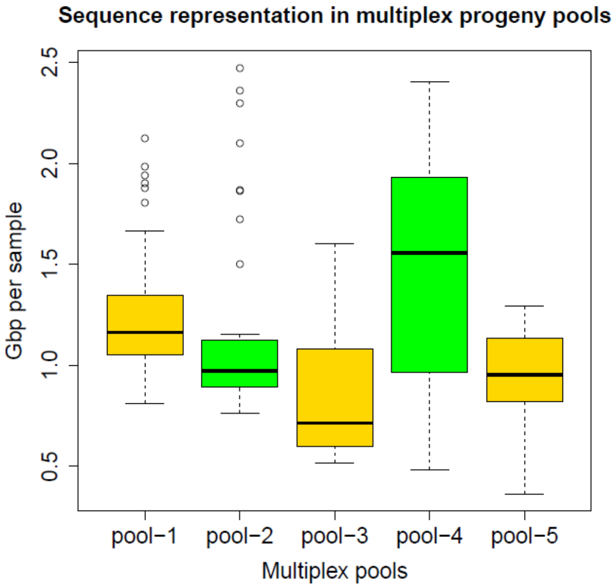

Figure S4 | Per-sample raw base pairs sequencing output on the five multiplexed runs used to collect low coverage (1X) progeny-wide reads.

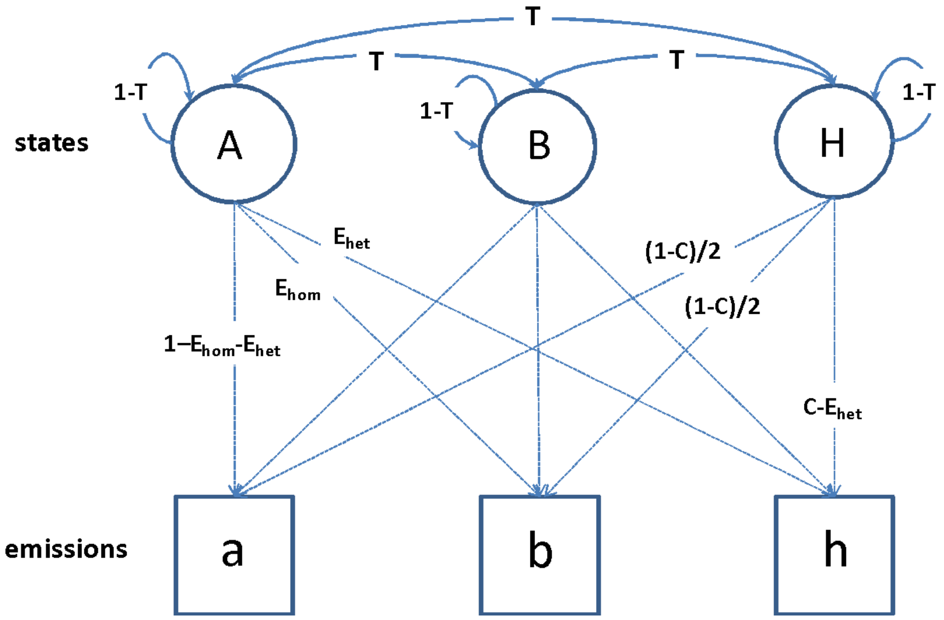

Figure S5 | Graphical representation of the HMM model developed for the decoding of most probable genotype paths given the observation of SNP calls at low coverage in an expected heterozygosity scenario. A, B, H are possible states of underlying sequence, while *a*, *b* and *h* represent low coverage calls obtained from Illumina reads. E_{het} and E_{hom} are the probability of false heterozygous calling for a given site. *C* is the probability of obtaining a heterozygous call at a given site by the presence of reads from both alleles. *T* is the probability of transition from one genotype to another (i.e. crossing-over events).

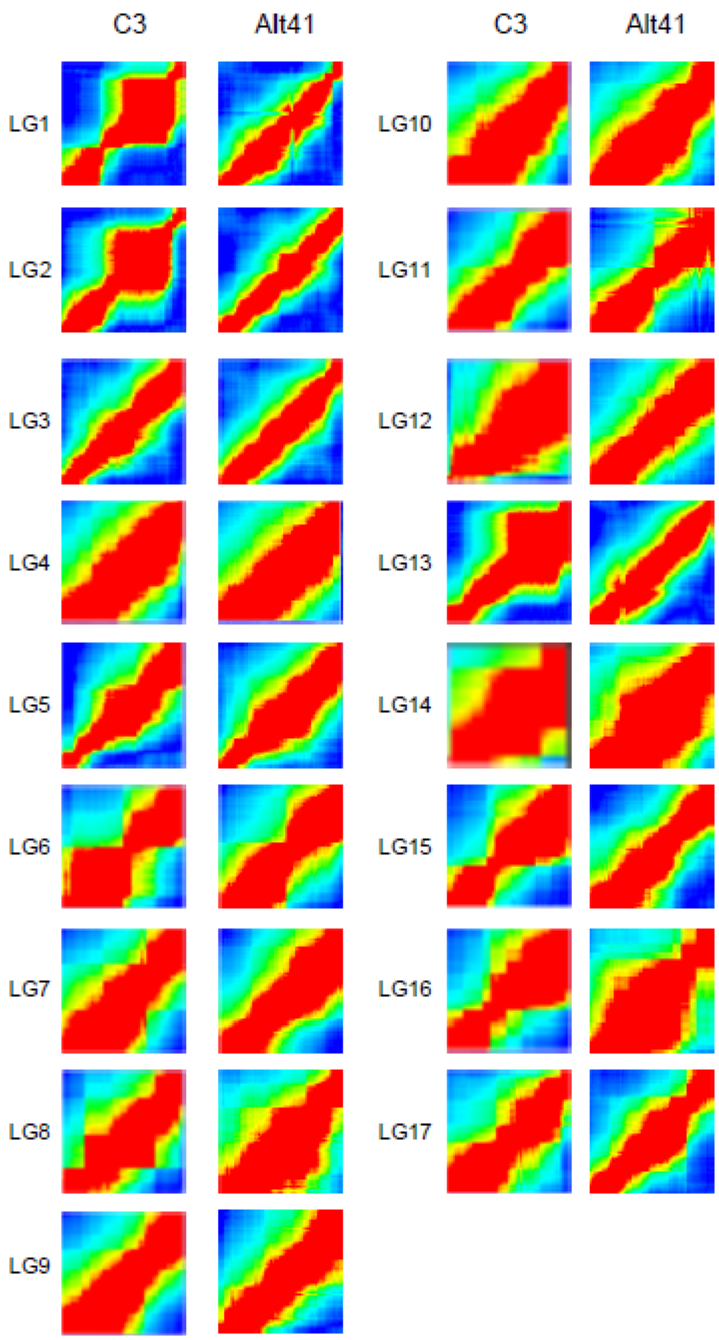

Figure S6 | Heat maps of the recombinant fraction (upper left sector) and LOD scores (bottom right sector) of linkage maps generated for Romanesco C3 and Altilis 41 parents. Red corresponds to lowest recombination fraction and highest LOD scores.

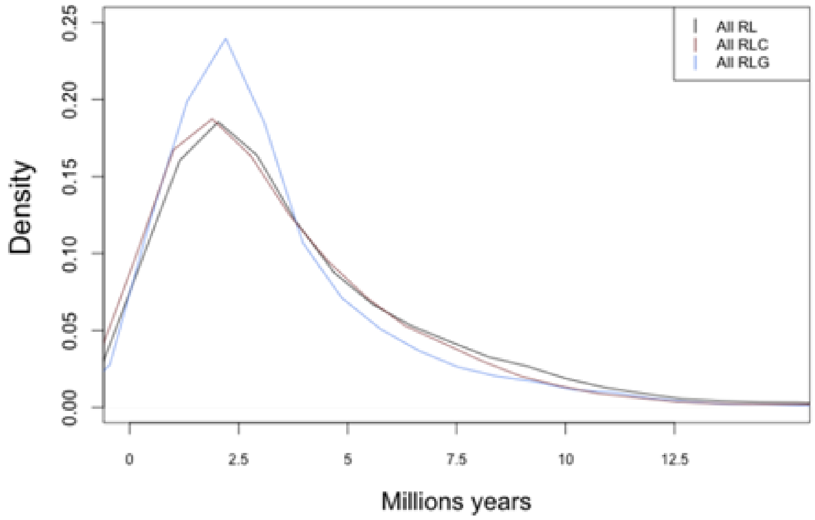

Figure S7 | Distribution of insertion ages of LTR elements

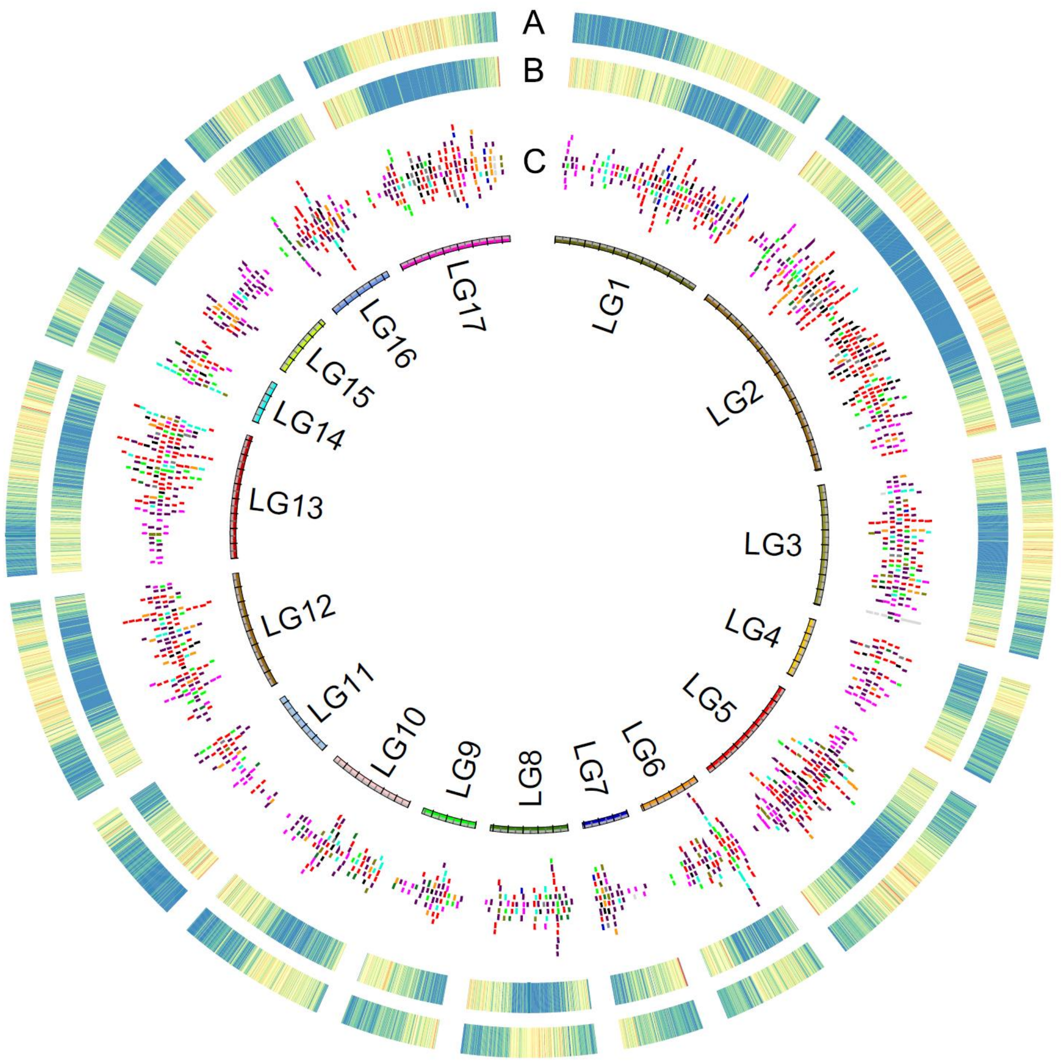

Figure S8 | Distribution of satellite sequences. Density of repeats (A) and genes (B) are report as heat map. Color-coded blocks (C) report non-consecutive occurrences of a given monomer along the sequence. Monomer are referred as in Supplementary Table S10; red: Cluster_0-10[96bp]; green: Cluster_1-4-8[136bp]; blue: Cluster_11-14[148bp]; violet: Cluster_12[103bp]; light-blue: Cluster_15[100bp]; dark-green: Cluster_16[82bp]; purple: Cluster_2[94bp]; black: Cluster_3[105bp]; olive: Cluster_5[111bp]; orange: Cluster_6-7[152bp]; grey: Cluster_9[106bp].

Supplementary Tables S1-S10

Table S1a | Alignment statistics obtained using GMAP. The x paramenter represents the amount of unaligned sequence that triggers search for the remaining sequence (default 40). It enables alignment of chimeric reads and may help with some non-chimeric reads. To turn off, - x was set to zero.

chimera margins (-x)	off	100	200	300	400
Unique aligments	29,349	31,459	33,729	34,721	35,138
Multiexonic aligments	1,893	1,817	1,827	1,871	1,880
No mapping	978	978	978	978	978
Putative chimeras	6,506	4,472	2,193	1,156	730
Mapping percentage	80.67%	85.93%	91.81%	94.49%	95.59%

Table S1b | **Results of CEGMA analysis.** Core eukaryotic genes (CEGs) are classified as four groups based on their average degree of conservation. Group 1 represents the least conserved genes of the reference set 248 CEGs, with the conservation degree increasing in subsequent groups through to group 4.Header legend: first column = number of 248 ultra-conserved CEGs present in genome; second column = percentage of 248 ultra-conserved CEGs present; third column = total number of CEGs present including putative orthologs; fourth column = average number of orthologs per CEG; fifth column = percentage of detected CEGS having more than 1 ortholog.

	CEGs found in assembly	248 CEGs set coverage (%)	# of putative CEGs	avg. # orthologs per CEG	CEGs w/ multiple orthologs (%)
**Complete alignments**	210	84.68	390	1.86	50.95
Group1	53	80.30	94	1.77	43.40
Group2	46	82.14	78	1.70	45.65
Group3	50	81.97	99	1.98	56.00
Group4	61	93.85	119	1.95	57.38
**Partial alignments**	239	96.37	534	2.23	66.95
Group1	63	95.45	123	1.95	55.56
Group2	54	96.43	114	2.11	61.11
Group3	58	95.08	142	2.45	75.86
Group4	64	98.46	155	2.42	75.00

Table S2 | *De novo* sequencing statistics for the two mapping parents.

library	reads	Gbp	raw coverage
C3-300bp	409,019,306	40.90	38
C3-600bp	111,342,540	11.13	10
**Total (C3)**	**520,361,846**	**52.03**	**48**
Alt41-300bp	365,978,250	36.60	34
Alt41-600bp	101,646,880	10.16	9
**Total (Alt41)**	**467,625,130**	**46.76**	**43**

Table S3 | Pseudomolecule statistics. In the last column the pseudomolecules and linkage group, as depicted in Portis *et al.,* 2009, relationships are reported; the nomenclature is from the C3 map.

Chr	length (bp)	avg. scaffold length	avg. GC content	non-AGCT bases	N° scaffolds	oriented scaffolds	oriented bp	oriented bp (%)	C3 (cM)	Alt41 (cM)	'C3' parental map
**1**	49707439	104647	34.40%	4211747	475	104	20760124	41.8%	146.35	182.62	C1
**2**	70339030	68623	35.80%	6880431	1025	174	26305745	37.4%	162.69	170.83	C2
**3**	40261365	111527	34.70%	3216535	361	68	17965994	44.6%	117.13	142.67	C3
**4**	20147618	119926	34.60%	1493262	168	23	7754853	38.5%	65.27	60.02	C4
**5**	37162917	110276	34.40%	3124327	337	50	11700443	31.5%	116.42	115.36	C5
**6**	20611051	89225	34.60%	2027785	231	36	7554461	36.7%	67.31	73.75	C6
**7**	15559887	170988	34.10%	1081502	91	27	8988908	57.8%	66.03	79.05	C7
**8**	25924184	112714	34.80%	2005747	230	38	8648700	33.4%	71.41	74.74	C8
**9**	18326914	106552	34.40%	1576491	172	43	9416779	51.4%	60.65	83.17	C9
**10**	29104343	100707	34.70%	2400099	289	52	12098280	41.6%	59.93	77.68	C10
**11**	22005725	196480	33.70%	1381011	112	34	11778224	53.5%	64.93	128.76	C11+C20
**12**	39646955	85816	35.10%	3702100	462	51	11008553	27.8%	70.28	91.45	C12
**13**	41505999	91222	34.70%	3530600	455	90	17212358	41.5%	120.78	140.35	C13+C18
**14**	14475048	113086	34.00%	1073474	128	25	5746158	39.7%	47.03	55.68	C14
**15**	21262825	172868	33.50%	1556777	123	45	14447054	67.9%	70.39	103.32	C15+C19
**16**	21914710	115951	34.60%	1799640	189	21	6168798	28.1%	67.82	65.64	C16
**17**	37690487	79516	35.90%	3591453	474	55	12083853	32.1%	85.16	103.06	C17
**Chr Total**	525646497	114713	34.59%	44652981	5322	936	2,1E+08	40%	-	-	-
**Unmapped**	199020768	24077	37%	25635496	8266	-	-	-	-	-	-
**Grand Total**	724667265	53091	36%	70288477	13588	-	-	-	-	-	-

Table S4 | Largest protein groups ranked by number of members.

Cluster Rank	Number Of Proteins	Predicted Function
1	364	LRR Kinases
2	308	LRR Kinases
3	287	EF-hand kinases
4	273	PPR Repeat
5	210	PPR Repeat
6	174	Cytochrome P450
7	119	UDP-glycosyltransferases
8	110	2OG-Fe(II) oxygenase superfamily
9	98	Ulp1 protease family
10	86	GDSL-like Lipase/Acylhydrolase
11	83	Ras family
12	78	Auxin responsive protein
13	77	Haem peroxidase superfamily
14	77	No apical meristem (NAM) protein
15	77	Cytochrome P450

Table S5 | Most common functional annotations in the artichoke predicted proteins.

Description	Number of proteins
Protein kinase domain	1357
Zinc finger	1041
Serine/Threonine protein kinases	898
Leucine Rich Repeat	607
PPR repeat family	598
Protein tyrosine kinase	498
EF-hand domain	424
Cytochrome P450	306
ATPase family associated with various cellular activities (AAA)	283
Ring finger domain	267

Table S6a | Statistical analysis of gene families per species.

Species	Gene Number	Genes in Families	Unclustered Genes	Number of Families	Unique Families	Genes in Unique Families	Average number of genes per family
A. thaliana	27,416	23,172	4,244	16,781	422	1,254	1.38
B. rapa	40,905	31,625	9,280	17,070	876	2,916	1.85
C. cardunculus	27,196	20,509	6,687	14,810	418	1,170	1.38
F. vesca	32,831	23,729	9,102	13,364	1,394	6,603	1.77
L. sativa	38,919	29,184	9,735	16,258	1,435	5,950	1.79
S. lycopersicon	34,727	24,907	9,820	14,307	1,118	4,743	1.74

Table S6b | Statistical analysis of expanded gene families in globe artichoke/lettuce and in globe artichoke in a six-species comparison. Legend: Atha=Arabidopsis; Brap=Brassica; Ccar=globe artichoke; Fves=Strawberry; Slyc=tomato

Prevalent expansion in globe artichoke

Clustering	Annotation	Group Name	Atha	Brap	Ccar	Fves	Lsat	Slyc	N° Species	Total Count	Mean Count	SD	CV	Adj X^2
-	Cystatin domain	Comp10193	0	0	33	1	1	1	4	36	9,00	13,24	1,47	1.17 E-24
Chr13	E-class P450 group I signature	Comp10249	0	0	27	0	1	4	3	32	5,33	10,73	2,01	2.11 E-3
-	Replication fact-A C-term domain (DUF23)	Comp10806	0	0	15	0	0	2	2	17	2,83	6,01	2,12	1.68 E-3
-	Glycosyltransferase family 10	Comp13550	0	0	8	0	1	0	2	9	1,50	3,21	2,14	NA

Prevalent expansion in globe artichoke/lettuce

Clustering	Annotation	Group Name	Atha	Brap	Ccar	Fves	Lsat	Slyc	N° Species	Total Count	Mean Count	SD	CV	Adj X^2
2 - Chr10	LRR	Comp10022	1	1	20	10	55	9	6	96	16,00	20,36	1,27	2.50 E-20
3 - Chr 5,6,9	Bulb-type mannose-specific lectin	Comp10125	0	0	19	8	18	2	4	47	11,75	8,77	0,75	1.04 E-7
1 - Chr 10	LRR	Comp10027	0	0	19	3	71	0	3	93	15,50	28,18	1,82	6.14 E-3
4- Chr 2,13,3,6	Pentatricopeptide (PPR)	Comp10088	0	0	36	0	21	0	2	57	9,50	15,46	1,63	5.66 E-3
1 – Chr 15	Pathogenesis-related protein Bet v I family	Comp10405	0	0	13	0	11	0	2	24	4,00	6,23	1,56	2.38 E-3

Table S6c | Globe artichoke specific genes and their clustering coordinates. The main biological process for each cluster is reported.

gene	strand	LG	LG coordinates	PHMMER (uniprotKB db)	e-value (phmmer)	PROCESS
Ccrd_v1.0_013577	-	1	38.330.947	Protein_ENHANCED_DOWNY	9,8E-39	Defense response to fungus
Ccrd_v1.0_013578	-	1	38.332.679	Protein_ENHANCED_DOWNY	1,3E-09	
Ccrd_v1.0_013579	-	1	38.333.957	Protein_ENHANCED_DOWNY	2,1E-17	
Ccrd_v1.0_013580	-	1	38.335.514	Protein_ENHANCED_DOWNY	4,8E-13	
Ccrd_v1.0_013581	-	1	38.335.793	Protein_ENHANCED_DOWNY	5E-23	
Ccrd_v1.0_013582	-	1	38.337.064	Protein_ENHANCED_DOWNY	2,6E-28	
Ccrd_v1.0_013583	-	1	38.342.744	Protein_ENHANCED_DOWNY	6,8E-14	
Ccrd_v1.0_013584	-	1	38.350.535	Protein_ENHANCED_DOWNY	4,5E-30	
Ccrd_v1.0_013585	-	1	38.352.862	Protein_ENHANCED_DOWNY	9,3E-57	
Ccrd_v1.0_013586	-	1	38.356.901	Protein_ENHANCED_DOWNY	1,1E-14	
Ccrd_v1.0_013587	-	1	38.357.345	Protein_ENHANCED_DOWNY	5,2E-28	
Ccrd_v1.0_013882	+	1	47.958.758	LRR_receptor-like_serine/threonine-protein	1,2E-103	Defense response signaling pathway
Ccrd_v1.0_013882	+	1	47.962.409	LRR_receptor-like_serine/threonine-protein	1,2E-103	
Ccrd_v1.0_013882	+	1	47.966.732	LRR_receptor-like_serine/threonine-protein	1,2E-103	
Ccrd_v1.0_013892	-	1	48.128.505	Receptor-like_protein_12	4,6E-107	
Ccrd_v1.0_013892	-	1	48.131.550	Receptor-like_protein_12	4,6E-107	
Ccrd_v1.0_013906	+	1	48.313.838	Myb-related_protein	1,2E-17	
Ccrd_v1.0_013907	+	1	48.325.308	Transcription_factor_MYB51	1,1E-12	
Ccrd_v1.0_013918	-	1	48.499.373	#N/D	#N/D	
Ccrd_v1.0_013927	-	1	48.653.548	Probable_acyl-activating_enzyme_peroxisomal	3,5E-22	
Ccrd_v1.0_013928	+	1	48.667.122	Probable_acyl-activating_enzyme_peroxisomal	1,2E-20	
Ccrd_v1.0_013947	-	1	48.983.694	Disease_resistance-like_protein	3E-13	
Ccrd_v1.0_013947	-	1	48.994.352	TMV_resistance_protein	3E-20	
Ccrd_v1.0_013952	+	1	49.085.948	Disease_resistance_protein	2,4E-16	
Ccrd_v1.0_013953	-	1	49.095.145	Putative_Myb_family_factor	8,6E-15	
Ccrd_v1.0_013954	+	1	49.100.100	Disease_resistance_protein	5,9E-26	
Ccrd_v1.0_013998	+	1	49.601.344	Syntaxin-61	1E-17	
Ccrd_v1.0_013998	+	1	49.606.095	Syntaxin-61	1E-17	
Ccrd_v1.0_009654	+	2	7.461.604	Transcription_factor_BIM1	0,0035	Transcription regulation
Ccrd_v1.0_009654	+	2	7.466.862	Transcription_factor_BIM1	0,0035	
Ccrd_v1.0_009658	+	2	7.547.756	unknown	0,42	
Ccrd_v1.0_009659	-	2	7.549.840	Tubulin_beta-2_chain	7,4E-172	
Ccrd_v1.0_009676	-	2	7.776.546	Transcriptional_activator	8,9E-48	
Ccrd_v1.0_009678	-	2	7.804.691	Myb-related_protein	1E-47	
Ccrd_v1.0_010683	+	2	61.492.858	Stearoyl-[acyl-carrier-protein]_9-desaturase_6	7,7E-159	Fatty Acids synthesis
Ccrd_v1.0_010683	+	2	61.497.880	Stearoyl-[acyl-carrier-protein]_9-desaturase_6	7,7E-159	
Ccrd_v1.0_010691	+	2	61.661.811	Stearoyl-[acyl-carrier-protein]_9-desaturase_6	8,6E-165	
Ccrd_v1.0_018157	+	5	6.441.006	Cyclin-dependent_kinase	5,2E-113	Cell cycle regulation
Ccrd_v1.0_018158	+	5	6.446.869	Cyclin-dependent_kinase	2,5E-114	
Ccrd_v1.0_018159	-	5	6.449.546	Cyclin-dependent_kinase	1,4E-129	
Ccrd_v1.0_001281	-	5	6.451.678	Cyclin-dependent_kinase	2,3E-100	
Ccrd_v1.0_018176	-	5	6.708.574	Cyclin-dependent_kinase	4,5E-114	
Ccrd_v1.0_018581	+	5	20.768.511	GTP-binding_nuclear_protein	5,2E-18	Protein transport
Ccrd_v1.0_018581	+	5	20.769.926	GTP-binding_nuclear_protein	5,2E-18	
Ccrd_v1.0_018581	+	5	20.771.685	GTP-binding_nuclear_protein	5,2E-18	
Ccrd_v1.0_018581	+	5	20.772.331	GTP-binding_nuclear_protein	5,2E-18	
Ccrd_v1.0_022377	-	6	11.215.709	Calreticulin	1,5E-227	Signal transduction
Ccrd_v1.0_022382	-	6	11.427.186	B2_protein	0,0018	
Ccrd_v1.0_022383	+	6	11.441.485	Putative_calmodulin-like_protein	0,000000063	
Ccrd_v1.0_022383	+	6	11.442.672	Putative_calmodulin-like_protein	0,000000063	
Ccrd_v1.0_022384	+	6	11.465.132	Calmodulin-like_protein	0,000000025	
Ccrd_v1.0_022580	+	6	16.109.439	Two-pore_potassium_channel	1,2E-30	ion TM transport
Ccrd_v1.0_022583	-	6	16.135.474	Two-pore_potassium_channel	1,4E-29	
Ccrd_v1.0_022582	+	6	16.138.823	Two-pore_potassium_channel	2,3E-23	
Ccrd_v1.0_022584	+	6	16.168.013	Two-pore_potassium_channel	5,2E-30	
Ccrd_v1.0_003123	+	9	7.792.930	Fibrillin-1	1,2E-79	Photooxidative stress
Ccrd_v1.0_003124	+	9	7.827.169	Fibrillin-3	4,6E-48	
Ccrd_v1.0_003138	-	9	8.112.291	Wall-associated_receptor_kinase	5E-14	
Ccrd_v1.0_003138	-	9	8.122.522	Wall-associated_receptor_kinase	5E-14	
Ccrd_v1.0_003138	-	9	8.125.063	Wall-associated_receptor_kinase	5E-14	
Ccrd_v1.0_003138	-	9	8.126.938	Wall-associated_receptor_kinase	5E-14	
Ccrd_v1.0_003139	-	9	8.158.833	Fibrillin-1	2,4E-75	
Ccrd_v1.0_003140	-	9	8.180.750	Wall-associated_receptor_kinase	4,6E-12	
Ccrd_v1.0_003141	-	9	8.203.319	Fibrillin-3	3,1E-85	
Ccrd_v1.0_003142	-	9	8.224.128	Fibrillin-3	3,3E-86	

gene	strand	LG	LG coordinates	PHMMER (uniprotKB db)	e-value (phmmer)	PROCESS
Ccrd_v1.0_021222	+	10	19.090.822	Protein_SUPPRESSOR_OF_CONSTITUTIVE_1	5,5E-36	Defence response
Ccrd_v1.0_023603	+	10	19.179.725	Probable_serine/threonine-protein_kinase	1,4E-99	
Ccrd_v1.0_021229	+	10	19.252.203	Receptor-like_protein_kinase	2,7E-53	
Ccrd_v1.0_021232	-	10	19.311.453	Probable_serine/threonine-protein_kinase	4,1E-26	
Ccrd_v1.0_021232	-	10	19.317.722	Probable_serine/threonine-protein_kinase	4,1E-26	
Ccrd_v1.0_016160	+	13	3.282.715	Putative_receptor-like_protein	4,8E-71	Defence response
Ccrd_v1.0_016161	+	13	3.286.163	Putative_receptor-like_protein	4,8E-59	
Ccrd_v1.0_016162	+	13	3.299.609	Receptor-like_protein_kinase	4E-67	
Ccrd_v1.0_016164	+	13	3.315.293	Receptor-like_protein_kinase_1	1,9E-68	
Ccrd_v1.0_016165	-	13	3.322.034	TMV_resistance_protein	1,4E-46	
Ccrd_v1.0_016170	+	13	3.370.130	Protein_SUPPRESSOR_OF_CONSTITUTIVE_1	1,7E-47	
Ccrd_v1.0_016173	+	13	3.412.192	unknown	0,09	
Ccrd_v1.0_016174	+	13	3.427.346	Protein_SUPPRESSOR_OF_CONSTITUTIVE_1	1,7E-47	
Ccrd_v1.0_016175	+	13	3.440.527	TMV_resistance_protein	3,1E-16	
Ccrd_v1.0_016175	+	13	3.440.527	TMV_resistance_protein	1,7E-16	
Ccrd_v1.0_016175	+	13	3.447.201	TMV_resistance_protein	1,7E-16	
Ccrd_v1.0_016175	+	13	3.447.202	TMV_resistance_protein	3,1E-16	
Ccrd_v1.0_016176	+	13	3.462.836	Protein_SUPPRESSOR_OF_CONSTITUTIVE_1	3,4E-43	
Ccrd_v1.0_016177	+	13	3.476.304	TMV_resistance_protein	8,4E-20	
Ccrd_v1.0_016178	+	13	3.482.883	TMV_resistance_protein	2,5E-15	
Ccrd_v1.0_016179	+	13	3.492.917	TMV_resistance_protein	1,4E-18	
Ccrd_v1.0_016196	-	13	3.799.430	RNA_polymerase_II_5-mediating_protein	0,0000022	
Ccrd_v1.0_016267	-	13	4.833.186	Serine/threonine-protein_kinase-like_protein	0,0000082	
Ccrd_v1.0_016291	-	13	5.184.329	Probable_receptor-like_protein	2,4E-69	
Ccrd_v1.0_008635	+	14	4.964.500	Chlorophyll_a-b_binding_16_chloroplastic	1,6E-162	response to light stimulus
Ccrd_v1.0_008642	-	14	5.121.295	Chlorophyll_a-b_binding_16_chloroplastic	6,6E-163	
Ccrd_v1.0_008644	+	14	5.146.900	ATP-dependent_Clp_protease_subunit_ClpA	0,0015	
Ccrd_v1.0_008645	-	14	5.155.501	Chlorophyll_a-b_binding_16_chloroplastic	2,1E-160	
Ccrd_v1.0_008646	-	14	5.168.538	Chlorophyll_a-b_binding_16_chloroplastic	2,8E-161	
Ccrd_v1.0_008647	-	14	5.220.185	Chlorophyll_a-b_binding_16_chloroplastic	3,4E-164	
Ccrd_v1.0_008736	-	14	8.288.381	Transcription_factor_MYB48	6,3E-14	Transcr. regulation
Ccrd_v1.0_008736	-	14	8.292.935	Transcription_factor_MYB48	6,3E-14	
Ccrd_v1.0_008737	-	14	8.293.175	Myb-related_protein_306_majus	1,3E-16	
Ccrd_v1.0_008997	-	14	14.153.985	Auxin_response_factor	2,7E-71	Auxin-activ. Signal. pathway
Ccrd_v1.0_008999	+	14	14.177.331	Auxin_response_factor	1,9E-83	
Ccrd_v1.0_009000	+	14	14.188.710	Auxin_response_factor	1,2E-57	
Ccrd_v1.0_001911	-	15	9.910.627	Auxin-responsive_protein_SAUR22	4,3E-30	Auxin-activated signaling pathway
Ccrd_v1.0_001912	-	15	9.919.876	Auxin-responsive_protein_SAUR22	4E-30	
Ccrd_v1.0_001914	-	15	9.929.794	Auxin-responsive_protein_SAUR21	5E-27	
Ccrd_v1.0_001921	-	15	10.026.525	Auxin-responsive_protein_SAUR19	5,4E-26	
Ccrd_v1.0_001923	+	15	10.029.056	Auxin-responsive_protein_SAUR22	3E-21	
Ccrd_v1.0_001922	-	15	10.029.639	Auxin-responsive_protein_SAUR21	1,5E-24	
Ccrd_v1.0_006281	-	16	4.067.666	Early_light-induced_protein_chloroplastic	1,6E-51	response to high light intensity
Ccrd_v1.0_006282	-	16	4.074.913	Early_light-induced_protein_chloroplastic	1,6E-48	
Ccrd_v1.0_006282	-	16	4.075.565	Early_light-induced_protein_chloroplastic	1,6E-48	
Ccrd_v1.0_006282	-	16	4.079.627	Early_light-induced_protein_chloroplastic	1,6E-48	
Ccrd_v1.0_006285	-	16	4.116.754	Early_light-induced_protein_chloroplastic	1E-50	
Ccrd_v1.0_006864	+	16	21.154.883	Protein_SUPPRESSOR_OF_CONSTITUTIVE_1	3,4E-22	Defence response
Ccrd_v1.0_006905	-	16	21.626.285	Protein_SUPPRESSOR_OF_CONSTITUTIVE_1	2,3E-37	
Ccrd_v1.0_006906	-	16	21.677.103	Protein_SUPPRESSOR_OF_CONSTITUTIVE_1	1,3E-33	
Ccrd_v1.0_006908	-	16	21.698.804	Protein_SUPPRESSOR_OF_CONSTITUTIVE_1	2,3E-36	
Ccrd_v1.0_019921	+	17	7.249.743	WAT1-related_protein	8,9E-51	Auxin-activated signaling pathway
Ccrd_v1.0_019923	+	17	7.281.844	WAT1-related_protein	1,8E-28	
Ccrd_v1.0_019925	+	17	7.311.521	WAT1-related_protein	5,4E-51	
Ccrd_v1.0_019926	+	17	7.327.575	WAT1-related_protein	5,9E-36	

Table S7 | Most abundant satellite sequences found in the assembly. Occurrences are considered for non-consecutive repeats. Representative sequences are up to 10% divergent from their cluster components.

Cluster name	monomer size	occurrences	representative sequence
Cluster_0-10	[96bp]	493	GGTTAGTACAACCAGAGGCTCTGGTCGTGAAGCCAGAAGCTCTGGAAGAGAGACC AGAGGGGCTCTGGTCCTGGATGTTGAATTATTTTCTAAGTATG
Cluster_2	[94bp]	477	TGTTCATAATGAAAGAACTATTGTTCACAAGTTGTTCATATTGTGTTCATATGTACA CTAGCTGCATATCTCAACATTGGCTCGCTCCTGAATG
Cluster_12	[103bp]	191	TGTGATAATGAATGTGATAATCATGTGATAATCACCGTTATACATCACTTGGGTTAT CGTTTTGGTTGATAAGTTAAATTTTTCGGTGTAATCATGTGATATGGG
Cluster_1-4-8	[136bp]	116	TTTTAGATTATTATCTTTATGAAATTACCAAAATGACCCTACAATTTGGAAATCGGT ATTTTAACAATACTTTACGTATTTGCTTTATAAAATTACCGAAATGACCATATGTCTT GGGAATTGGTATCTAAAATTTA
Cluster_3	[105bp]	100	TTCTATTTCATCGGCCGCTGGTTCAACATCCATACTTAGAAAAAATAATGGATTTCT CGTCCATCGGTCCTCTGGTTCCACATACATACTTAGCAAACCAATGGAA
Cluster_15	[100bp]	94	ACGATCCATTAAAGCCCAATACGATCCATTAAAGCCCAATACGATCCATTAAATCCC AATACGATCCATTAAACCCAATAACGATCCACTAAAGTCCAAT
Cluster_6-7	[152bp]	89	TTGAAGGTCTTTTGAACGAGTTGAGAAAGCCTACATGTAATGTTTTGACTACTAGA AAGTCAAACCGTAGGTTTAGAGGAGTTTCATATGACTTTAAAACTCTAAGTGTAAC GGTTTGACTACTTGAAAGTCAAACCGTAGGTGTGAATGAAG
Cluster_16	[82bp]	46	CTCAGACTTTTATACTGGTATGTCTTACAAGTCTGGAAGACAACTTTGAATTTTAAC TGAGGAAAAAGGGTTCAGACTTATC
Cluster_5	[111bp]	44	CCATTTTCGGCGGATTTCCGGCAGAAAATTAAAAAATTTTAAAAAAAAAGAATAAA CAATTTTCCGGCGGATTTCCGGCGGATAATTAAATAATTTTTCTAAAAAAATTTA
Cluster_9	[106bp]	39	AAGAAGAACCAGAGGATCGATGGACGGATTGCTTAGTATTTTTCTAAGTGTAGAA GTTGAGCCAGAGGCTCTGGTCTTTGCAACCAGAAGCTCTGGTAAGTGCCCA
Cluster_11-14	[148bp]	14	GATTTAATAGTTGTCGTTGTGTTGTCAAATCCGGTCATAAACTTTCCATGGACTTGT TAAATCGTCTCTGACATTATTCGTCAGTAAAGCTTAAAAGATTCATCCTGAAACCCT GACCTATAACCCCTACGAATGTACTATCGCCCCCTAC

Table S8 | Chromosome-wise distribution of perfect SSRs. Mononucleotide = min. 15 times; dinucleotides = min. 8 times; trinucleotides = min. 5 times; tetra/penta/hexa-nucleotides = min. 4 times, allowing for only one mismatch. For compound repeats, the maximum default interruption (spacer) = 100bp.

Chr	Mono	Di	Tri	Tetra	Penta	Hexa	Total	Compound	Imperfect
**1**	772	9,637	1,345	872	366	368	13,360	2,000	18,311
**2**	747	10,834	1,993	893	359	386	15,212	2,228	21,071
**3**	566	8,660	1,206	774	302	335	11,843	1,894	16,354
**4**	304	4,553	507	349	171	155	6,039	942	8,163
**5**	523	8,476	1,081	688	288	275	11,331	1,894	15,557
**6**	284	4,683	517	414	161	133	6,192	1,061	8,478
**7**	267	4,059	376	367	122	140	5,331	932	7,268
**8**	369	5,894	752	537	244	189	7,985	1,333	11,040
**9**	270	4,502	456	349	145	167	5,889	943	7,949
**10**	434	5,797	781	447	184	227	7,870	1,253	10,762
**11**	447	5,410	629	508	208	191	7,393	1,135	10,064
**12**	474	7,436	1,087	651	217	238	10,103	1,546	13,842
**13**	568	8,227	1,066	718	279	334	11,192	1,737	15,326
**14**	241	4,002	342	347	136	113	5,181	915	7,034
**15**	414	5,456	574	519	178	153	7,294	1,208	9,926
**16**	295	4,921	594	416	165	141	6,532	1,088	8,936
**17**	366	6,066	1,077	502	201	211	8,423	1,256	11,670
**Unplaced**	1,052	20,777	5,526	1,391	576	715	30,037	3,917	42,162
**Total**	**8,393**	**129,390**	**19,909**	**10,742**	**4,302**	**4,471**	**177,207**	**27,282**	**243,913**

Table S9 | MiRNA genes identified in the globe artichoke genome and number of putative coding regions for each miRNA type.

miRNA	number of putative coding regions	miRNA	number of putative coding regions
miR1030	1	miR414	31
miR1039	1	miR419	3
miR1078	1	miR477	3
miR1127	1	miR479	2
miR1155	1	miR5021	7
miR1171	1	miR5058	1
miR1435	2	miR5161	1
miR1436	1	miR5174	4
miR1439	1	miR5181	1
miR1446	3	miR5185	1
miR156/157	24	miR5203	2
miR159/319	3	miR530	3
miR160	7	miR5523	1
miR164	3	miR5543	1
miR166	16	miR5653	2
miR167	3	miR5658	54
miR168	1	miR5821	1
miR169	17	miR6103	3
miR171	3	miR6104	6
miR172	11	miR6105	910
miR1857	1	miR6106	2
miR1862	1	miR6107	3
miR1888	1	miR6108	1495
miR2079	1	miR6109	131
miR2102	1	miR6110	161
miR2105	1	miR6111	1
miR2923	1	miR6112	1301
miR390	1	miR6114	2
miR393	4	miR6115	480
miR394	8	miR6116	15
miR395	8	miR6117	43
miR396	10	miR7741	1
miR397	3	miR7757	1
miR399	5	miR837	1
miR403	1	miR845	6
miR407	2	miR902	1
miR408	1	**TOTAL**	4832

Table S10 | miRNAs classification based on miRNA presence in RR, NRR, MIX regions.

**Elements only in "RR": 22**	**Elements only in "NRR": 30**	**"MIXED": 21**
miR1030 (1 locus) (*Ppt*)	miR1039 (1 locus) (*Ppt*)	miR156/157 (24 loci, 2RR) (*Htu*, *Sly*, *Stu*, *Nta*, *Vvi*, *Ath*, *Osa*, *Bdi*, *Ppt*)
miR1127 (1 locus) (*Bdi*)	miR1078 (1 locus) (*Ppt*)	miR159/319 (3 loci, 1RR) (*Htu*, *Sly*, *Stu*, *Nta*, *Vvi*, *Ath*, *Osa*, *Bdi*, *Ppt*)
miR1171 (1 locus) (*Cre*)	miR1155 (1 locus) (*Cre*)	miR166 (16 loci, 10RR) (*Sly*, *Stu*, *Nta*, *Vvi*, *Ath*, *Osa*, *Bdi*, *Pab*, *Ppt*)
miR1436 (1 locus) (*Osa*)	miR1435 (2 loci) (*Osa*)	miR169 (17 loci, 2RR) (*Sly*, *Stu*, *Nta*, *Vvi*, *Ath*, *Osa*, *Bdi*)
miR1439 (1 locus) (*Osa*)	miR1446 (3 loci) (*Nta*)	miR393 (4 loci, 1RR) (*Htu*, *Stu*, *Vvi*, *Ath*, *Osa*, *Bdi*)
miR2105 (1 locus) (*Osa*)	miR160 (7 loci) (*Htu*, *Sly*, *Stu*, *Nta*, *Vvi*, *Ath*, *Osa*, *Bdi*, *Pab*, *Ppt*)	miR394 (8 loci, 6RR) (*Nta*, *Vvi*, *Ath*, *Osa*, *Bdi*)
miR2923 (1 locus) (*Osa*)	miR164 (3 loci) (*Stu*, *Nta*, *Vvi*, *Ath*, *Osa*, *Bdi*)	miR396 (10 loci, 2RR) (*Stu*, *Nta*, *Vvi*, *Ath*, *Osa*, *Bdi*, *Pab*)
miR419 (3 loci) (*Ppt*)	miR167 (3 loci) (*Sly*, *Stu*, *Nta*, *Vvi*, *Ath*, *Osa*, *Bdi*, *Ppt*)	miR407 (2 loci, 1RR) (*Ath*)
miR5161 (1 locus) (*Osa*)	miR168 (1 locus) (*Sly*, *Nta*, *Vvi*, *Ath*, *Osa*, *Bdi*)	miR414 (31 loci, 27RR) (*Ath*, *Osa*)
miR5174 (4 loci) (*Bdi*)	miR171 (3 loci) (*Htu*, *Sly*, *Stu*, *Nta*, *Vvi*, *Osa*, *Bdi*)	miR5021 (7 loci, 6RR) (*Ath*)
miR5181 (1 locus) (*Bdi*)	miR172 (11 loci) (*Sly*, *Stu*, *Nta*, *Vvi*, *Ath*, *Osa*, *Bdi*)	miR5658 (54 loci, 50RR) (*Ath*)
miR5185 (1 locus) (*Bdi*)	miR1857 (1 locus) (*Osa*)	miR6104 (6 loci, 5RR)
miR5203 (2 loci) (*Bdi*)	miR1862 (1 locus) (*Osa*)	miR6105 (910 loci, 869RR)
miR530 (3 loci) (*Htu*, *Stu*, *Osa*)	miR1888 (1 locus) (*Ath*)	miR6107 (3 loci, 2RR)
miR5543 (1 locus) (*Osa*)	miR2079 (1 locus) (*Ppt*)	miR6108 (1495 loci, 1183RR)
miR5653 (2 loci) (*Ath*)	miR2102 (1 locus) (*Osa*)	miR6109 (131 loci, 117RR)
miR5821 (1 locus) (*Osa*)	miR390 (1 locus) (*Stu*, *Nta*, *Vvi*, *Ath*, *Osa*, *Bdi*, *Ppt*)	miR6110 (161 loci, 150RR)
miR6106 (2 loci)	miR395 (8 loci) (Stu, *Nta*, *Vvi*, *Ath*, *Osa*, *Bdi*, *Pab*)	miR6112 (1301 loci, 1195RR)
miR6111 (1 locus)	miR397 (3 loci) (*Sly*, *Stu*, *Bdi*, *Pab*)	miR6115 (480 loci, 414RR)
miR7741 (1 locus) (*Bdi*)	miR399 (5 loci) (*Sly*, *Stu*, *Nta*, *Vvi*, *Ath*, *Osa*, *Bdi*)	miR6117 (43 loci, 40RR)
miR7757 (1 locus) (*Bdi*)	miR403 (1 locus) (Htu, *Vvi*, *Ath*)	miR845 (6 loci, 3RR) (*Vvi*, *Ath*)
miR902 (1 locus) (*Ppt*)	miR408 (1 locus) (*Stu*, *Nta*, *Osa*, *Bdi*, *Ppt*)	
	miR477 (3 loci) (*Stu*, *Nta*)	
	miR479 (2 loci) (*Stu*, *Nta*, *Vvi*)	
	miR5058 (1 locus) (*Bdi*)	
	miR5523 (1 locus) (*Osa*)	
	miR6103 (3 loci)	
	miR6114 (2 loci)	
	miR6116 (15 loci)	
	miR837 (1 locus) (*Ath*)	

Supplementary data

Genome completeness

The GMAP aligner (Wu *et al.,* 2005) was adopted to assess the completeness of the globe artichoke genome using the existing ESTs of *C. cardunculus*. Different analyses were conducted varying the -x parameter (x = off, 100, 200, 300 and 400), which enables alignment of chimeric reads, and may help with some non-chimeric reads. The total number of unigenes found was obtained by considering both unique aligments and multi-exonic aligments. It reached a maximum of 35,138 (-x = 400) and 33,729 (-x = 200) with a mapping percentage of 95.59% and 91.81%, respectively. The non-mapping reads (978) remained constant even when varying the -x parameters. All the GMAP outputs are reported in the Supplementary table S1a.

The CEGMA (Core Eukaryotic Genes Mapping Approach; Parra *et al.,* 2007), pipeline was adopted to assess the completeness of the globe artichoke genome. A set of 248 core proteins that are present in a wide range of taxa, thereby are highly conserved, have been used to identify their exon-intron structures in genomic sequences. The lack of complete alignments for less conserved ortholog groups are probably due to divergence (CEGMA), while partial alignments confirm an even representation of different ortholog groups, indicating a gene space coverage ranging between 95% to 98.5 % (Supplementary table S1b).

Plastid and mitochondrial sequences

We searched the globe artichoke WGS assembly for fragments with similarity to chloroplast DNA. The search was conducted on a filtered dataset which did not include the scaffolds which were previously attributed to any linkage groups in order to avoid false positive hits to chloroplast hits genes resident in the genome). Using BlastN (*e*-value ≤10−5; Altschul 1997), we compared the filtered assembly to a draft of the *C. cardunculus* chloroplast genome (KM035764, Curci *et al.,* 2015). This search revealed few scaffolds with significant similarity, ranging from about 1 kb to 82 Kb in length that consisted primarily of chloroplast DNA. Chloroplast-like scaffolds were filtered out from the draft, using BLASTn data related to pairs presenting more than 95% identity and nucleotide coverage exceeding 10%. We did not detect a complete assembly of the plastid genome among the globe artichoke scaffolds, while many occurrences of plastid-related sequences were observed within the mapped scaffolds of nuclear DNA. The amount of mitochondrial sequence was surveyed as well and a few scaffolds showed similarity to *Nicotiana tabacum* mitochondrial DNA (NC_006581), none of them contained significant portions of the mitochondrial genome. The lack of organellar scaffolds is probably a consequence of ALLPATHs LG ignoring reads with unexpectedly high read depth.

miRNAs analysis in globe artichoke

Overall 4,832 genomic regions (Supplementary file S1) distributed on the 17 pseudomolecules (on average 284 regions for pseudomolecule) were predicted to be candidate miRNA-coding loci belonging to 73 different miRNAs (Supplementary table S7). Of these, 4,478 regions (93%) represented only 6 *C. cardunculus*-specific miRNAs (namely miR6105, miR6108, miR6109, miR6110, miR6112, miR6115).

Evidence of expression (RNAseq) was found for 122 out of 4,832 predicted putative miRNA-coding regions (2.5%; >10 reads). Considering publicly available unigene (Scaglione *et al*., 2012) and EST (Lai *et al*., 2009) datasets, evidence of expression was found for 882 miRNA-related genomic regions for the former, while ESTs gave evidence of expression for 789 miRNA-related genomic regions. Note that because of the presence of several miRNA putative paralogs, different loci were declared as expressed

based on the same EST or transcripts data. Consequently, the number of different associated transcripts and ESTs was lower: 188 transcripts (3.89%; 79 of them are functionally annotated) and 105 EST (2.17%) respectively. By considering as evidence of expression the combination of RNAseq data (> 10 reads reads) aligned) and at least one EST or one transcript, 119 miRNA-related regions (out of the 122 RNAseq-positives, >10 reads) were identified as putatively expressed (97.5%). The summary of the expression data for each putative miRNA-coding genomic region is reported in Supplementary file S2.

Supplementary literature

- Altschul, S.F., *et al.* Gapped BLAST and PSI-BLAST: a new generation of protein database search programs. Nucleic Acids Res. 25:3389-3402 (1997).
- Curci, P., De Paola, D., Danzi, D., Vendramin, G. & Sonnante, G. Complete Chloroplast Genome of the multifunctional Crop Globe Artichoke and Comparison with Other Asteraceae. Plos One 10 (2015).
- Lai, Z. *et al.* Genomics of Compositae weeds: EST libraries, microarrays, and evidence of introgression. American Journal of Botany, 99(2), 1–10 (2012).
- Parra, G., Bradnam, K. & Korf, I. CEGMA: a pipeline to accurately annotate core genes in eukaryotic genornes. Bioinformatics 23, 1061-1067 (2007).
- Scaglione, D. *et al.* Large-scale transcriptome characterization and mass discovery of SNPs in globe artichoke and its related taxa. Plant Biotech. J. 10, 956-969 (2012).
- Wu T.W., & Watanabe, C.K.. GMAP: a genomic mapping and alignment program for mRNA and EST sequences. Bioinformatics 21: 1859-187 (2005).

Supplementary file list

Supplementary file S1 – List of the 1,170 globe artichoke specific genes.
Supplementary file S2 – MiRNAs genomic coordinates and features.
Supplementary file S3 – Annotation of miRNA targets (a) and their GO enrichment analysis (b).
